# N^6^‐Methyladenosine Regulates Cilia Elongation in Cancer Cells by Modulating HDAC6 Expression

**DOI:** 10.1002/advs.202408488

**Published:** 2024-11-13

**Authors:** Yalan Rui, Haisheng Zhang, Kangning Yu, Shiyao Qiao, Chenglin Gao, Xiansong Wang, Weifeng Yang, Gholamreza Asadikaram, Zigang Li, Kun Zhang, Jianxin Peng, Jiexin Li, Junming He, Hongsheng Wang

**Affiliations:** ^1^ Guangdong Provincial Key Laboratory of New Drug Design and Evaluation State Key Laboratory of Anti‐Infective Drug Discovery and Development School of Pharmaceutical Sciences Sun Yat‐sen University Guangzhou 510006 China; ^2^ Endocrinology and Metabolism Research Center Institute of Basic and Clinical Physiology Sciences Kerman University of Medical Sciences Medical University Campus Kerman 7616913555 Iran; ^3^ Institute of Systems and Physical Biology Shenzhen Bay Laboratory Shenzhen 518067 China; ^4^ The Second Affiliated Hospital of Chengdu Medical College China National Nuclear Corporation 416 Hospital Chengdu Seventh People's Hospital Affiliated Cancer Hospital of Chengdu Medical College School of Biological Sciences and Technology Chengdu Medical College Chengdu 610500 China; ^5^ Department of Hepatobiliary Surgery Guangdong Province Traditional Chinese Medical Hospital Guangzhou 510120 China

**Keywords:** acetylated α‐tubulin, cilia length, HDAC6, m^6^A, cervical cancer

## Abstract

Primary cilia are microtubule‐based organelles that function as cellular antennae to address multiple metabolic and extracellular cues. The past decade has seen significant advances in understanding the pro‐tumorigenic role of N^6^‐methyladenosine (m^6^A) modification in tumorigenesis. Nevertheless, whether m^6^A modification modulates the cilia dynamics during cancer progression remains unclear. Here, the results show that m^6^A methyltransferase METTL3 regulates cilia length in cancer cells via HDAC6‐dependent deacetylation of axonemal α‐tubulin, thereby controlling cancer development. Mechanically, METTL3 positively regulates the translation of HDAC6 in an m^6^A‐dependent manner, while m^6^A methylation of A3678 in the coding sequence (CDS) of HDAC6 ameliorates its translation efficiency via facilitating the binding with YTHDF3. The upregulation of HDAC6 induced by METTL3 over‐expression is capable of inhibiting cilia elongation and acetylation of α‐tubulin, thereby shortening cilia length and accelerating the progression of cervical cancer both in vitro and in vivo. Collectively, depletion of METTL3‐mediated m^6^A modification leads to abnormally elongated cilia via suppressing HDAC6‐dependent deacetylation of axonemal α‐tubulin, ultimately attenuating cell growth and cervical cancer development.

## Graphical abstract

METTL3 positively regulates the translation of HDAC6 in an m^6^A‐dependent manner. Briefly, m^6^A methylation of A3678 in CDS ameliorates its translation efficiency by facilitating the binding with YTHDF3. Depletion of METTL3‐mediated m^6^A modification promotes cilia elongation and acetylation of α‐tubulin. The abnormally elongated cilia length attenuates the progression of cervical cancer both in vitro and in vivo.

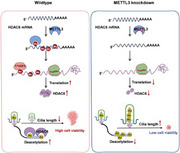

## 1 Introduction

Primary cilia are solitary and microtubule‐based non‐motile organelles originating from the cell surface, capable of sensing the surrounding extracellular stimuli.^[^ ^1^ ^]^ Given their special antenna‐like structure, emerging studies have broadened our understanding of the complex mechanisms that control biogenesis and the function of primary cilia in cellular processes during the development and homeostasis of tissues and organs, especially in cancers.^[^ ^2^, ^3^, ^4^ ^]^ For example, disruption of the ciliary GTPase ARL13B has been reported to inhibit medulloblastoma formation, while KLHDC8A, as a novel molecular target of cancer stem cells, is elucidated to facilitate the assembly of primary cilia, ultimately providing new insights in augmenting effects against glioblastoma proliferation.^[^ ^5^ ^]^ The primary cilium has been shown to be responsible for regulating the Hedgehog (Hh) signaling pathway, thereby controlling the progression of a spectrum of human tumors.^[^ ^6^ ^]^ Defects in primary structure and function, especially changes in ciliary length, may lead to various diseases, including cancer.^[^ ^7^ ^]^ Nowadays, the viewpoints on primary cilia in mediating cancer pathogenesis are highly divergent, which is elucidated to serve as both tumor suppressors and oncogenic drivers in different cancers and within tumor subtypes.^[^ ^2^ ^]^ The concrete mechanism dissected how primary cilia mediate tumorigenesis remains ambiguous.

It has been reported that tubulin acetylation plays a key role in cilia assembly, disassembly, and length regulation, while the removal of acetyl groups from lysine residues of proteins is mainly regulated by histone deacetylases (HDACs).^[^ ^8^, ^9^ ^]^ Based on their sequence homology, the 18 members of the HDAC family are mainly categorized into four classes: class I (HDAC1, 2, 3, and 8), class IIa (HDAC4, 5, 7, and 9), class IIb (HDAC6 and 10), class III (sirtuin family: sirt1‐sirt7), class IV (HDAC11).^[^ ^10^ ^]^ According to the research, HDAC2 depletion was reported to account for the recovery of primary cilia formation in pancreatic ductal adenocarcinoma.^[^ ^11^ ^]^ Additionally, HDAC3 and HDAC8 have been reported to participate in cilia regulation.^[^ ^12^ ^]^ It is worth noting that with the advancement of ciliary biology, HDAC6, as a unique cytoplasmic member of the HDAC family, has long been emphasized as a key regulator of ciliary disassembly and elongation.^[^ ^13^ ^]^ In terms of its deacetylase activity, deacetylation of α‐tubulin or cortactin by HDAC6 is predominantly recognized to relate to microtubule destabilization and ciliary disassembly.^[^ ^14^, ^15^ ^]^

N^6^‐methyladenosine (m^6^A) methylation is the most prevalent and dominant internal transcriptional modification in mammalian RNA.^[^ ^16^ ^]^ m^6^A modification is dynamic and reversible, which can be manipulated by m^6^A “writers”, “erasers”, and “readers”.^[^ ^17^ ^]^ Since m^6^A modification regulates the splicing, translation, transport, degradation, stability, and processing of RNA, dysregulation of m^6^A modification is commonly associated with disease development, including cancers.^[^ ^18^ ^]^ Methyltransferase‐like 3 (METTL3), as a core subunit of the complex m^6^A methyltransferase, has been documented to play a pivotal role in tumorigenesis.^[^ ^19^ ^]^ Meanwhile, METTL3 was reported to deteriorate papillary thyroid cancer progression by disturbing the Hedgehog signaling pathway.^[^ ^20^ ^]^ Additionally, METTL3‐dependent m^6^A methylation plays an essential role in cilium‐related symptoms, including fertility and osteoporosis.^[^ ^21^, ^22^ ^]^ Increasing evidence links the association between METTL3 and primary cilia.^[^ ^20^ ^]^ However, how METTL3 participates in the cilia assembly and elongation remains unclear.

In present study, METTL3 was identified as an authentic manipulator of cilia assembly and elongation associated with cancer development. Mechanically, m^6^A methylation of A3678 in the coding sequence (CDS) of HDAC6 ameliorates its translation efficiency by facilitating the binding with YTHDF3. It reveals the essential role of the METTL3‐HDAC6 axis in regulating cilia assembly and elongation via modulating deacetylation of axonemal α‐tubulin, thereby providing a novel insight into therapeutic vulnerabilities in cancer treatment.

## 2 Results

### 2.1 METTL3 Suppresses the Ciliary Elongation in Cancer Cells

**Figure 1:**
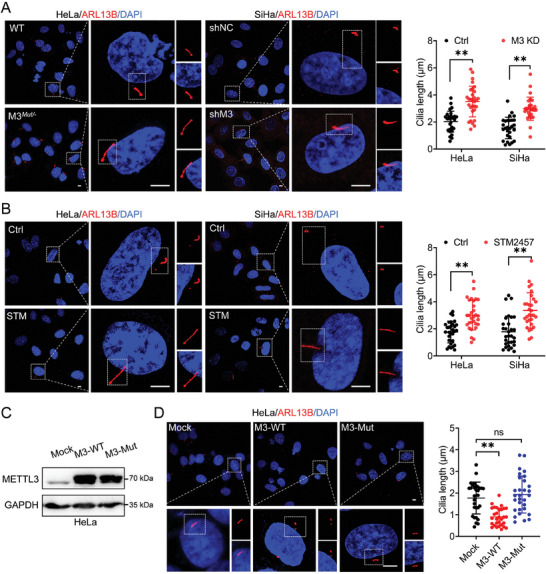
METTL3 suppresses the ciliary elongation in cancer cells. A) HeLa/SiHa cells with METTL3 depletion were cultured under serum starvation for 72 h. Primary cilia were visualized by immunofluorescence using anti‐ARL13B antibody (red) and nuclei were stained with DAPI (blue). Cilia length was measured and presented as bar graphs (*right*) (Scale bar, 5 µm). B) HeLa/SiHa cells treated with STM2457 (2 µg ml^−1^) were cultured under serum starvation for 72 h. Primary cilia were visualized by immunofluorescence using anti‐ARL13B antibody (red), and nuclei were stained with DAPI (blue). Cilia length was measured and presented as bar graphs (*right*) (Scale bar, 5 µm). C,D) HeLa wild‐type (WT) cells were transfected with PPB‐METTL3 (M3‐WT), PPB‐METTL3 mutant (M3‐Mut), or empty vector PPB (Mock), respectively. C) Expression of METTL3 was confirmed by western blot assay. D) Primary cilia were visualized by immunofluorescence analysis. Cilia length was measured and presented as bar graphs (*right*) (Scale bar, 5 µm). Data are presented as mean ± SD from three independent experiments. *p < 0.05, **p < 0.01, ***p < 0.001, ns, not significant, by Student's *t*test between two groups, and by one‐way ANOVA followed by Bonferroni's test for multiple comparisons‐.

To validate whether METTL3 plays a role in ciliary regulation in cancer cells, the depletion of METTL3 was achieved in both HeLa and SiHa cells for further investigation (Figure S1, Supporting Information). The assembly of primary cilia was triggered in cervical cancer cells by serum starvation as previously reported.^[^ ^23^ ^]^ Herein, ARL13B is used as a marker for primary cilia, while γ‐tubulin is used as a marker for the basal body. As shown in **Figure** **1**A, silencing METTL3 displayed a nonnegligible effect on the cilia elongation of cancer cells. Notably, the cilia length of ciliated cells in METTL3*^Mut/−^* HeLa cells was significantly increased compared to HeLa wild‐type (WT) cells (Figure 1A). Similarly, abnormally elongated cilia were also increased in SiHa cells upon METTL3 depletion (Figure 1A). STM2457 is a highly potent inhibitor of METTL3 that suppresses its transferase activity.^[^ ^24^ ^]^ After treating either HeLa or SiHa cells with STM2457, significantly enhanced elongation of cilia length in ciliated cells was observed (Figure 1B). It indicated that METTL3 negatively regulated the cilia elongation in cancer cells.

We further checked whether the cilia elongation is affected by the m^6^A catalytic activity of METTL3. To pursue this hypothesis, overexpression of METTL3 wildtype (M3‐WT) or its catalytically inactive METTL3 mutant DA (D395A; M3‐Mut) was performed as described in a previous study (Figure 1C).^[^ ^25^ ^]^ The results showed that overexpression of WT METTL3, but not of METTL3 DA mutant, reduced cilia length in HeLa cells (Figure 1D). Moreover, compared with HeLa cells transfected with empty vector (Mock), there was no significant change in either proportion or length of ciliated cells transfected with METTL3 DA mutant constructs with serum deprivation (Figure 1D). Collectively, these results suggest that METTL3 is inversely correlated with cellular ciliation, which is dependent on its m^6^A catalytic activity.

### 2.2 HDAC6 is Involved in METTL3‐regulated Cilia Elongation

**Figure 2:**
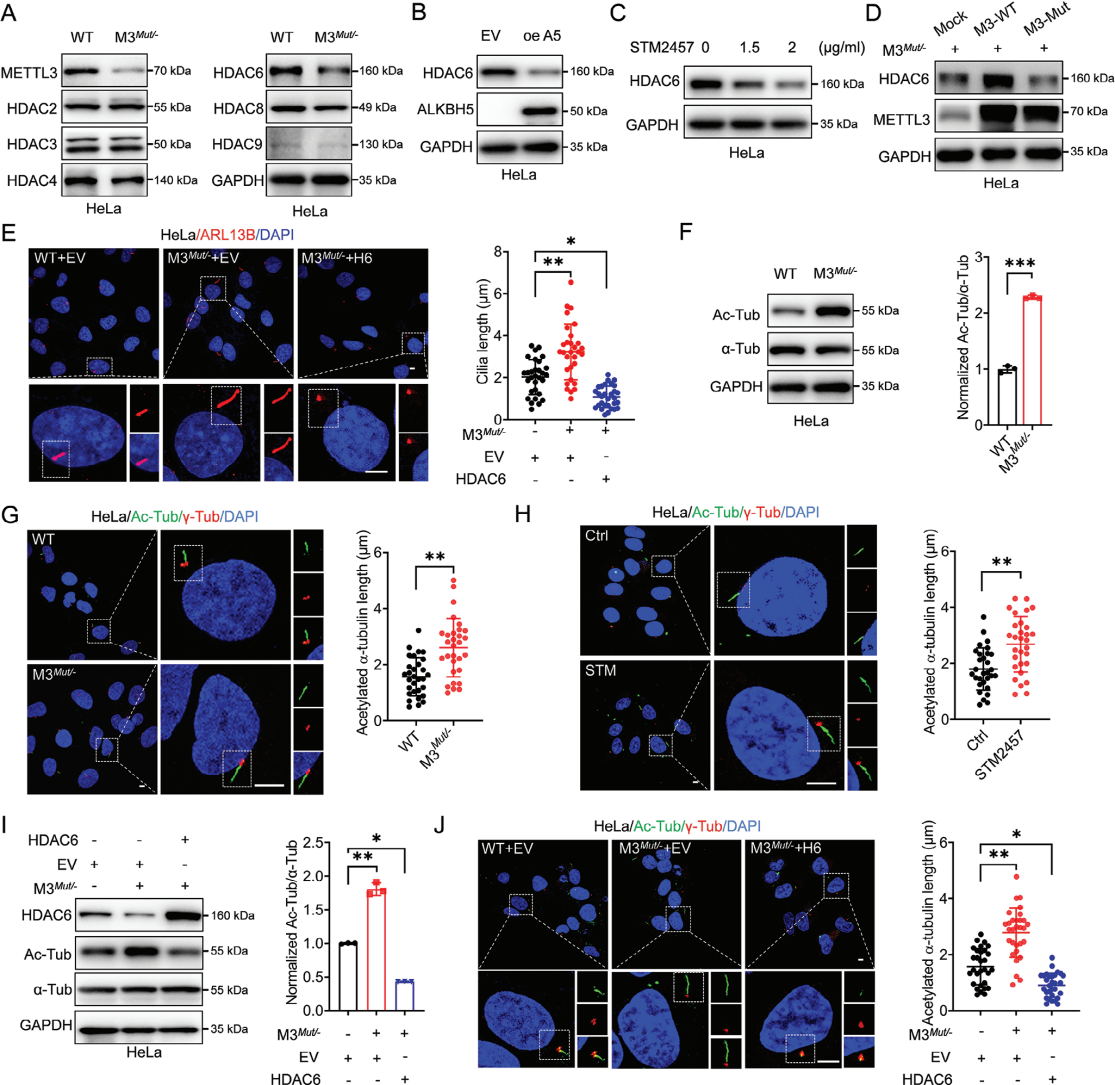
HDAC6 is involved in METTL3‐regulated cilia elongation. A) The protein levels of six HDACs belonging to three different classes were systematically examined in WT or METTL3*^Mut/‐^* HeLa cells by western blot analysis. B) The protein expression of HDAC6 in HeLa cells transfected with vector control or ALKBH5 plasmid was measured by western blot analysis. C) The protein levels of HDAC6 in HeLa WT cells treated with different concentrations of STM2457 (1.5, 2 µg ml^−1^) for 48 h were measured by western blot analysis. D) The expression of HDAC6 in METTL3*^Mut/‐^* HeLa cells transfected with the empty vector PPB (mock), PPB‐METTL3 (M3 wild type [WT]), or PPB‐METTL3 mutant (M3 mutant) was detected by western blot analysis. E) The length of cilia in WT or METTL3*^Mut/−^* HeLa cells transfected with empty vector (EV, pcDNA3.1) or HDAC6 constructs (pcDNA3.1‐HDAC6‐3 × HA) for 72 h was measured. Primary cilia were visualized by immunofluorescence using anti‐ARL13B antibody (red), and nuclei were stained with DAPI (blue). Cilia length was measured and presented as bar graphs (*right*) (Scale bar, 5 µm). F) The relative ratio of acetylated α‐tubulin/α‐tubulin (Ac‐Tub/α‐Tub) in WT or METTL3*^Mut/−^* HeLa cells was confirmed by western blot analysis. G) WT or METTL3*^Mut/−^* HeLa cells were cultured under serum starvation for 72 h. Primary cilia were visualized by immunofluorescences using anti‐acetylated‐α‐tubulin (ac‐α‐tubulin) antibody (green) and the basal body using γ‐tubulin antibody (red), and nuclei were stained with DAPI (blue). The acetylated α‐tubulin length was measured and presented as bar graphs (*right*) (Scale bar, 5 µm). H) The acetylated α‐tubulin length of cilia in HeLa cells treated with DMSO or STM2457 (2 µg ml^−1^) for 48 h were examined. Primary cilia were visualized by immunofluorescences using anti‐ac‐α‐tubulin antibody (green) and the basal body using γ‐tubulin antibody (red), and nuclei were stained with DAPI (blue). The acetylated α‐tubulin length was measured and presented as bar graphs (*right*) (Scale bar, 5 µm). I) The relative ratio of ac‐Tub/α‐Tub in WT or METTL3*^Mut/‐^* HeLa cells transfected with empty vector (EV, pcDNA3.1) or HDAC6 constructs (pcDNA3.1‐HDAC6‐3 × HA) for 48 h was measured by western blot analysis. J) The acetylated α‐tubulin length of cilia in WT or METTL3*^Mut/‐^* HeLa cells transfected with empty vector (EV, pcDNA3.1) or HDAC6 constructs (pcDNA3.1‐HDAC6‐3×HA) for 72 h were measured. Primary cilia were visualized by immunofluorescence using anti‐ac‐α‐tubulin antibody (green) and the basal body using γ‐tubulin antibody (red), and nuclei were stained with DAPI (blue). The acetylated α‐tubulin length was measured and presented as bar graphs (*right*) (Scale bar, 5 µm). Data are presented as mean ± SD from three independent experiments. *p < 0.05, **p < 0.01, ***p < 0.001, ns, not significant, by Student's *t*‐test between two groups and by one‐way ANOVA followed by Bonferroni's test for multiple comparisons‐.

As acknowledged, acetylation plays an essential role in cilia assembly, we thus aimed to investigate whether HDACs are involved in regulating METTL3‐dependant cilia dynamics. Protein levels of six HDACs belonging to three different classes were tested upon METTL3 silencing. The results showed that HDAC6 was significantly decreased in METTL3 knockdown HeLa cells (**Figure** **2**A). Similar results were obtained in sh‐METTL3 SiHa cells (Figure S2A, Supporting Information). In addition, HDAC6 expression levels were significantly decreased in both HeLa and SiHa cells overexpressing m^6^A demethylase ALKBH5 (Figure 2B; Figure S2B, Supporting Information), suggesting that HDAC6 expression in cervical cancer cells is closely associated with m^6^A modification. To verify this suggestion, HDAC6 protein levels treated with different concentrations of STM2457 were examined. As a result, expression of HDAC6 in HeLa or SiHa cells was down‐regulated in a dose‐dependent manner after STM2457 treatments (Figure 2C; Figure S2C, Supporting Information). We further performed a rescue experiment in METTL3*^Mut/−^* HeLa cells transfected with WT METTL3 or catalytically inactive METTL3 mutant. The results demonstrated that overexpression of WT METTL3, rather than overexpression of the catalytically inactive METTL3 mutant, dramatically enhanced the HDAC6 levels in METTL3*^Mut/−^* HeLa cells (Figure 2D). Our data implied that the expression of HDAC6 was anchored by METTL3 in an m^6^A‐dependent manner.

HDAC6 has been reported to be associated with cilia length regulation.^[^ ^26^ ^]^ This suggestion was confirmed by immunofluorescent assay, showing that overexpression of HDAC6 in METTL3*^Mut/−^* HeLa cells successfully impaired the increase of cellular cilia length induced by METTL3 deficiency (Figure 2E; Figure S2D, Supporting Information). Notably, this impairment was more severe when compared to HeLa WT cells, which may result from the abundant existence of HDAC6 in cells (Figure S2D, Supporting Information). Together, the above results suggested that HDAC6 played a role in the METTL3‐suppressed cilia length regulation.

As reported, HDAC6 is endogenous in the cytoplasm of HeLa cells (Figure S2E, Supporting Information). α‐tubulin, as one of the key components of cilia, is recognized as a substrate of HDAC6.^[^ ^27^ ^]^ We thus hypothesized that HDAC6 may participate in the METTL3‐regulated cilia assembly and elongation via deacetylating α‐tubulin. First, western blot analysis showed that the acetylation level of tubulin significantly enhanced in METTL3 knockdown cells (Figure 2F; Figure S2F, Supporting Information). The acetylated α‐tubulin was essential to the cilia assembly since the continuous linear signals of acetylated α‐tubulin were detected from the basal body of cilia (γ‐tubulin) (Figure 2G; Figure S2G, Supporting Information). Remarkably, the length of acetylated α‐tubulin was significantly enhanced in METTL3 knockdown cells (Figure 2G; Figure S2G, Supporting Information). Second, administration of STM2457 elongated the acetylated α‐tubulin signals in both HeLa and SiHa cells (Figure 2H; Figure S2H, Supporting Information). Last, silencing METTL3 in HeLa cells significantly enhanced the level of acetylated α‐tubulin, which corresponds to the abnormally elongated acetylated α‐tubulin signals in cilia (Figure 2I,J). These observations were reversed when overexpressing HDAC6 in METTL3 knockdown cells (Figure 2I,J). Together, it indicated that HDAC6 was involved in METTL3‐suppressed cilia assembly and elongation via deacetylating α‐tubulin.

### 2.3 METTL3 Regulates Translation of HDAC6 via YTHDF3

**Figure 3:**
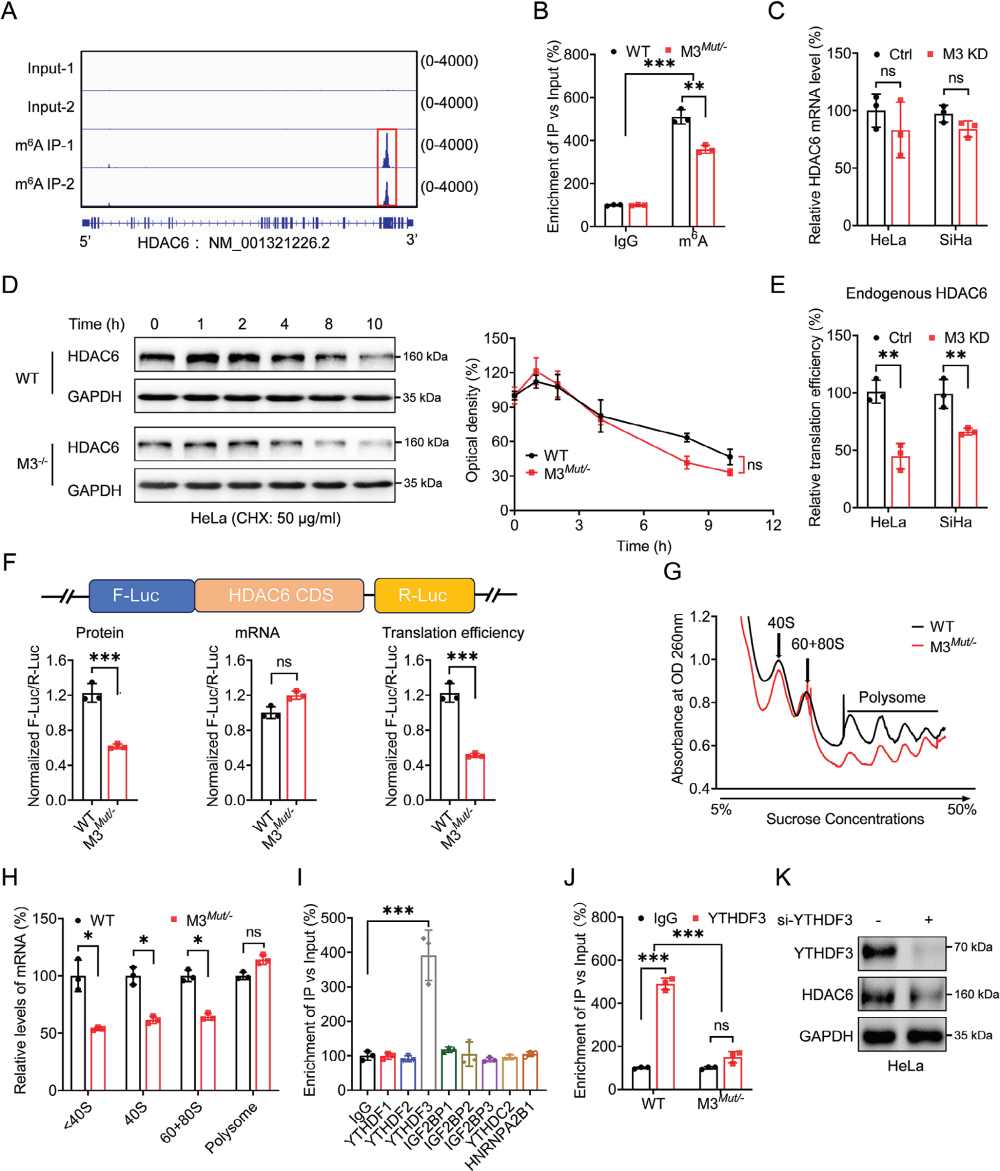
METTL3 regulates the translation of HDAC6 via YTHDF3. A) m^6^A peak was enriched in CDS of HDAC6 genes from m^6^A RIP‐seq data. B) m^6^A RIP‐qPCR analysis of HDAC6 mRNA in WT or METTL3*^Mut/‐^* HeLa cells. C) The relative mature‐mRNA levels of HDAC6 in METTL3‐knockdown HeLa or SiHa cells were measured. D) After treating WT or METTL3*^Mut/−^* HeLa cells with 50 µg ml^−1^ CHX for the indicated time, the expression of HDAC6 was detected by western blot analysis (*left*) and quantitatively analyzed (*right*). E) The translation efficiency of endogenous HDAC6 was checked by normalizing HDAC6 protein levels to the corresponding mRNA abundance. F) WT or METTL3*^Mut/−^* HeLa cells were transfected with pmirGLO‐HDAC6‐CDS‐WT reporter for 24 h, respectively. The protein, mRNA, and translation efficiency were determined. G) The polysome profiling of WT or METTL3*^Mut/−^* HeLa cells was analyzed. H) The mRNA levels of HDAC6 in non‐translational fraction (< 40S), translation initiation fraction (including 40S, 60S ribosomes, 80S monosomes) and polysome fractions in WT or METTL3*^Mut/−^* HeLa cells were analyzed by qRT‐PCR. I) RIP‐qPCR analysis of HDAC6 mRNA in HeLa WT cells using antibodies against YTHDF1–3, IGF2BP1–3, YTHDC2, and HNRNPA2B1. J) Binding between YTHDF3 and HDAC6 mRNA in WT or METTL3*^Mut/−^* HeLa cells was detected by RIP‐qPCR. K) Expression levels of HDAC6 in WT or METTL3*^Mut/−^* HeLa cells silencing YTHDF3 were detected by western blot assay. Data are presented as mean ± SD from three independent experiments. *p < 0.05, **p < 0.01, ***p < 0.001, ns, not significant, by Student's *t*‐test between two groups and by one‐way ANOVA followed by Bonferroni's test for multiple comparisons.

Since HDAC6 expression is regulated by m^6^A level, and HDAC6 mRNA contains m^6^A peaks in our previous sequencing data (**Figure** **3**A), we hypothesized that HDAC6 expression is regulated by m^6^A modification. Thus, we suppose that m^6^A modification may exert distinct effects on HDAC6 expression. m^6^A‐RIP‐qPCR results confirmed that HDAC6 mRNA was m^6^A modified. The m^6^A enrichment of HDAC6 mRNA was ≈5.03±0.34‐fold in HeLa WT cells, while it drastically declined to an average of 3.76±0.17‐fold in METTL3*^Mut/−^* cells (Figure 3B), indicating reversible m^6^A modification in HDAC6 mRNA. To explore how m^6^A regulates HDAC6 expression, the results showed that knock‐down of METTL3 neither affects the expression level of HDAC6 mRNA (Figure 3C) nor the sub‐cellular localization (Figure S3A, Supporting Information), as well as the protein stability of HDAC6 (Figure 3D). Instead, the translation efficiency of endogenous HDAC6, depicted by the relative ratio of protein content to mRNA abundance, was obviously diminished accompanied by METTL3 downregulation (Figure 3E). We further constructed a pmirGLO‐HDAC6 luciferase reporter containing both F‐Luc and HDAC6 CDS regions (Figure S3B, Supporting Information). Dual‐luciferase assay showed that the translation efficiency of HDAC6 in METTL3*^Mut/−^* HeLa cells was significantly down‐regulated compared to WT cells (Figure 3F). Together, it indicated that m^6^A modulated the translation process of HDAC6.

Ribosome profiling was performed to undermine the molecular mechanism of m^6^A modification in HDAC6 translation. In line with the previous report,^[^ ^28^ ^]^ the global translation process was suppressed in METTL3 knockdown HeLa (Figure 3G) and SiHa cells (Figure S3C, Supporting Information). We then detected HDAC6 mRNA levels in the non‐translational fraction (< 40S), translation initiation fraction (including 40S and 60S ribosomes, 80S monosomes), and translation active polysomes (>80S) between control and METTL3 knockdown cells. The RT‐qPCR results displayed that the translation initiation fraction (including 40S, 60S ribosomes, and 80S monosomes) of METTL3 knockdown cells, rather than translation active polysomes (>80S), was visibly lower than the corresponding control group of HeLa or SiHa cells (Figure 3H; Figure S3D, Supporting Information). It hints that METTL3 and m^6^A may be involved in mediating the translation initiation of HDAC6.

To investigate how m^6^A regulates the translation of HDAC6, m^6^A “readers” related to translation regulation, including YTHDF1/2/3, YTHDC2, HNRNPA2B1, and IGF2BP1/2/3 were examined.^[^ ^29^, ^30^ ^]^ RIP‐qPCR revealed that HDAC6 mRNA was significantly enriched in the YTHDF3‐IP group of HeLa cells (Figure 3I; Figure S3E, Supporting Information), hinting that YTHDF3 recognizes m^6^A in HDAC6 mRNA and regulates its translation. Moreover, the binding between YTHDF3 and HDAC6 mRNA was substantially diminished in cells with METTL3 deletion (Figure 3J). In addition, the results showed that si‐YTHDF3 could suppress the protein expression of HDAC6 in HeLa cells (Figure 3K; Figure S3F, Supporting Information). Together, these results indicated that YTHDF3 participated in translating HDAC6 mRNA.

### 2.4 METTL3 Regulates Translation of HDAC6 at A3678 via an m^6^A‐Dependent Manner

**Figure 4:**
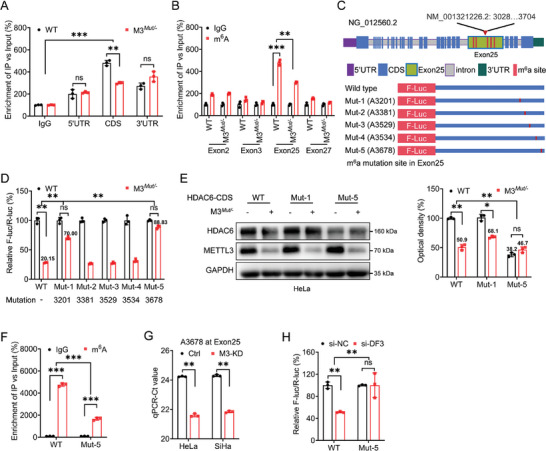
METTL3 regulates the translation of HDAC6 at A3678 via an m^6^A‐dependent manner. A) The m^6^A in 5′UTR, CDS, and 3′UTR of HDAC6 in WT or METTL3*^Mut/−^* HeLa cells were analyzed by m^6^A‐RIP‐qPCR using fragmented RNA. B) The m^6^A of HDAC6 in WT or METTL3*^Mut/−^* HeLa cells were analyzed by m^6^A‐RIP‐qPCR using fragmented RNA to analyze the m^6^A enrichment of different exons. C) Schematic representation of mutated A–C) exon 25 of HDAC6 mRNA of pmirGLO vector to investigate the role of m^6^A in modulating HDAC6 expression. D) The relative luciferase activity of F‐Luc/R‐Luc of pmirGLO‐HDAC6‐CDS‐WT or pmirGLO‐exon25‐Mut‐1/‐2/‐3/‐4/‐5 in WT or METTL3*^Mut/−^* HeLa cells were determined. E) WT or METTL3*^Mut/−^* HeLa cells were transfected with vector control, pcDNA3.1‐HDAC6‐CDS‐WT, and pcDNA3.1‐HDAC6‐exon25‐Mut‐1/‐5 for 24 h, the expression of HDAC6 was checked by western blot analysis (*left*) and quantitatively analyzed (*right*). F) HeLa cells were transfected with pmirGLO‐exon25‐WT or pmirGLO‐exon25‐Mut‐5 for 24 h, and the m^6^A in F‐Luc exon fusion mRNA was analyzed by m^6^A‐RIP‐qPCR. G) The threshold cycle (Ct) of qPCR showed SELECT results for detecting the m^6^A site at A3678 of HDAC6 CDS in HeLa or SiHa cells. H) The relative luciferase activity of F‐Luc/R‐Luc was measured in WT or METTL3*^Mut/−^* HeLa cells co‐transfected with si‐YTHDF3 combined with pmirGLO‐HDAC6‐CDS‐WT or pmirGLO‐exon25‐Mut‐5, respectively, for 48 h. Data are presented as mean ± SD from three independent experiments. *p < 0.05, **p < 0.01, ***p < 0.001, ns, not significant, by Student's *t‐*test between two groups, and by one‐way ANOVA followed by Bonferroni's test for multiple comparisons.

Next, we investigated which m^6^A site(s) is responsible for the HDAC6 translation regulation. In line with the MeRIP‐seq data, m^6^A‐RIP‐qPCR results confirmed that the m^6^A modification occurred in the CDS region of HDAC6 mRNA, but not in the 5′UTR or 3′UTR regions (**Figure** **4**A). To achieve the effective screening of particular m^6^A sites responsible for HDAC6 regulation, the authoritative m^6^A prediction tool SRAMP (http://www.cuilab.cn/sramp) was used. The results suggested that the potential m^6^A modification may occur in exon 2, exon 3, exon 25, and exon 27 (Figure S4, Supporting Information). Accordingly, specific qPCR primers targeting those exon regions were designed. m^6^A‐RIP‐qPCR using fragmented RNA indicated that exon 25 was the most m^6^A‐modified, and this modification was significantly reduced in METTL3 knockdown HeLa cells (Figure 4B).

There are five predicted sites located in exon 25 with high m^6^A confidence based on SRAMP results (Figure S4, Supporting Information). On one hand, we mutated the pmirGLO‐HDAC6‐CDS reporter to construct Mut‐1/2/3/4/5 (base “A” to “C”) accordingly (Figure 4C). Dual‐luciferase assays showed that both Mut‐1 (A3201C) and Mut‐5 (A3678C) successfully attenuated the reduction in fluorescence activity caused by METTL3 knockdown (Figure 4D). On the other hand, exogenous HDAC6 mutants with A3201C (Mut‐1) and A3678C (Mut‐5) were constructed, respectively. Western blot results showed that only the expression of Mut‐5 was suppressed in HeLa cells (Figure 4E). m^6^A‐RIP‐qPCR confirmed a lower m^6^A enrichment in HDAC6 Mut‐5 group (Figure 4F). Collectively, our data indicated that A3678 of HDAC6 CDS was responsible for the METTL3‐regulated expression of HDAC6.

We further validated whether A3678 is the critical site involved in the m^6^A/YTHDF3‐regulated translation of HDAC6. First, the “SELECT” method for the single site detection of m^6^A validated the reversible modification of A3678 in endogenous HDAC6 mRNA (Figure 4G).^[^ ^31^ ^]^ Second, dual‐luciferase assays using pmirGLO‐HDAC6‐CDS‐WT/Mut‐5 were performed in HeLa WT cells silencing YTHDF3. The results showed that the luciferase activity of pmirGLO‐HDAC6‐CDS‐WT was successfully reduced after silencing YTHDF3, while such deletion was not observed in HeLa cells transfected with pmirGLO‐HDAC6‐CDS‐Mut‐5 (Figure 4H). Together, our results indicated that m^6^A modification in A3678 participated in the translation of HDAC6.

### 2.5 Targeted Demethylation of HDAC6 by dm^6^ACRISPR Disturbs Cilia Elongation

**Figure 5:**
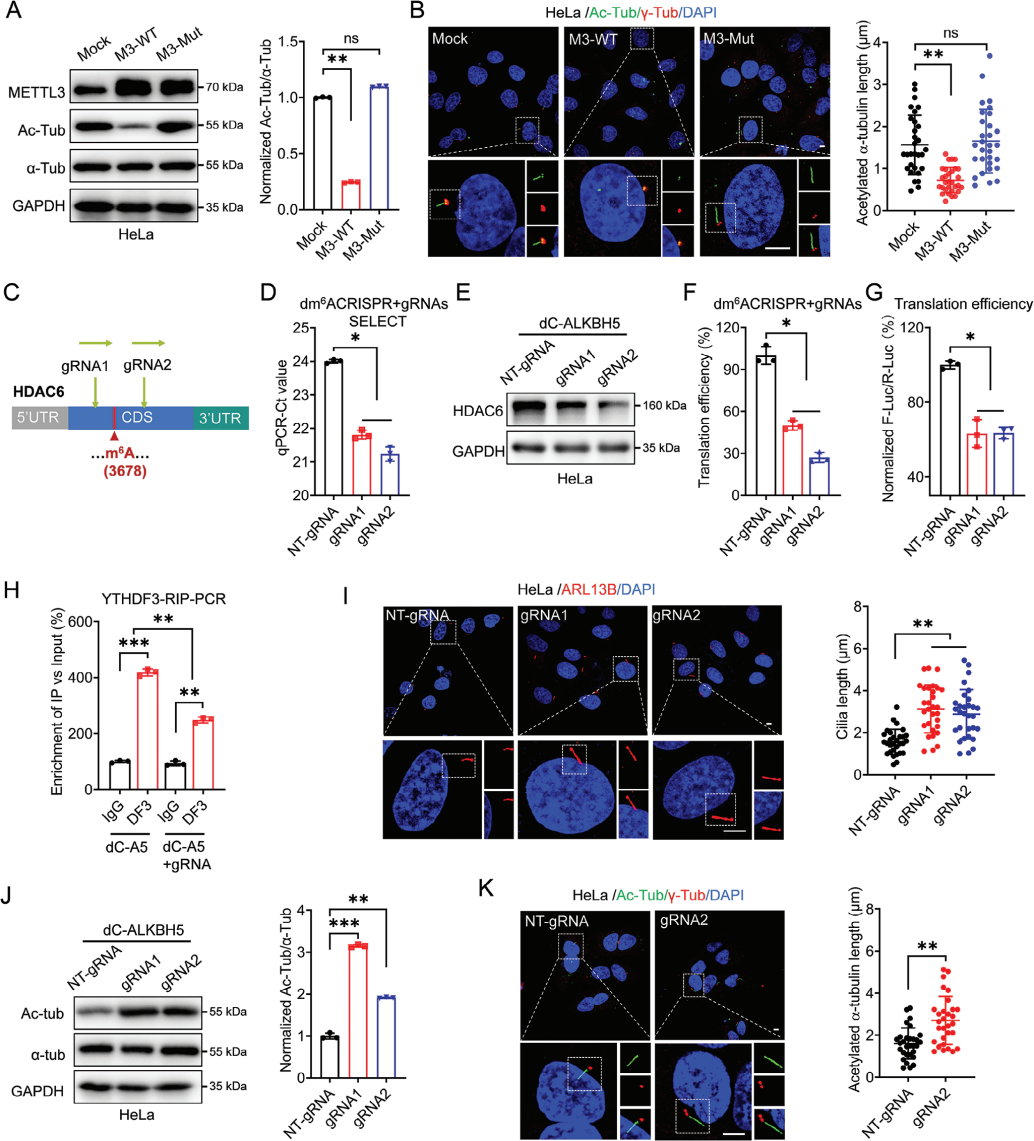
Targeted demethylation of HDAC6 by dm^6^ACRISPR disturbs cilia elongation. A) The expression of ac‐tubulin in HeLa WT cells transfected with METTL3 wildtype (M3‐WT), METTL3‐Mut (M3‐Mut), or its empty vector (Mock) was confirmed by western blot assay using anti‐METTL3 antibody, respectively. B) HeLa cells were transfected with METTL3, METTL3‐Mut, or empty plasmid, respectively. Primary cilia were visualized by immunofluorescences using anti‐ac‐α‐tubulin antibody (green) and the basal body using γ‐tubulin antibody (red), and nuclei were stained with DAPI (blue). The acetylated α‐tubulin length was measured and presented as bar graphs (*right*) (Scale bar, 5 µm). C) Schematic representation of positions of m^6^A site within HDAC6 mRNA and the regions targeted by two gRNAs, respectively. D) The threshold cycle (Ct) of qPCR revealed SELECT results for detecting m^6^A site in HDAC6 mRNA in HeLa cells transfected with dCas13b‐ALKBH5 combined with non‐targeted gRNA negative control (NT‐gRNA) or gRNA1/2, respectively, for 24 h. E) The expression of HDAC6 in HeLa cells transfected with dCas13b‐ALKBH5 combined with gRNA negative control or gRNA1/2, respectively, for 24 h was checked by western blot analysis. F) The translation efficiency of endogenous HDAC6 was checked by normalizing HDAC6 protein levels to the corresponding mRNA abundance in HeLa cells transfected with dCas13b‐ALKBH5 combined with gRNA negative control or gRNA1/2, respectively, for 24 h. G) WT or METTL3*^Mut/−^* HeLa cells were co‐transfected with pmirGLO‐HDAC6‐CDS‐WT reporter and dCas13b‐ALKBH5 combined with gRNA negative control or gRNA1/2, for 24 h. The translation efficiency was determined. H) RIP‐qPCR analysis of HDAC6 mRNA in HeLa cells transfected with dCas13b‐ALKBH5 combined with gRNA negative control or gRNA2, respectively, for 48 h by using the antibody against YTHDF3. I) The length of cilia in HeLa cells transfected with dCas13b‐ALKBH5 combined with gRNA negative control or gRNA1/2, respectively, for 72 h were measured. Primary cilia were visualized by immunofluorescences using anti‐ARL13B antibody (red) and nuclei were stained with DAPI (blue). Cilia length was measured and presented as bar graphs (*right*) (Scale bar, 5 µm). J) The expression of ac‐tubulin, tubulin, and GAPDH in HeLa cells transfected with dCas13b‐ALKBH5 combined with gRNA negative control or gRNA1/2, respectively, for 48 h, was checked by western blot analysis (*left*) and quantitatively analyzed (*right*). K) The length of cilia in HeLa cells transfected with dCas13b‐ALKBH5 combined with gRNA negative control or gRNA2, respectively, for 72 h were measured. Primary cilia were visualized by immunofluorescences using anti‐ac‐α‐tubulin antibody (green) and the basal body using γ‐tubulin antibody (red), and nuclei were stained with DAPI (blue). The acetylated α‐tubulin length was measured and presented as bar graphs (*right*) (Scale bar, 5 µm). Data are presented as mean ± SD from three independent experiments. *p < 0.05, **p < 0.01, ***p < 0.001, ns, not significant, by Student's *t*‐test between two groups, and by one‐way ANOVA followed by Bonferroni's test for multiple comparisons.

It has been verified that METTL3 regulates the expression of HDAC6 in an m^6^A‐dependent manner, it therefore probably similarly controls the acetylation of tubulin. As expected, overexpression of METTL3 drastically suppressed the acetylation level of tubulin in HeLa cells, while its catalytically inactive METTL3 mutant DA (D395A) failed to trigger such a decline (**Figure** **5**A). Consistently, overexpression of METTL3 WT, rather than METTL3 mutant, significantly disturbed the cilia elongation in HeLa cells, which was represented by a visible decrease in cilia length (Figure 5B). Meanwhile, similar results were also observed in SiHa cells (Figure S5A, Supporting Information). It suggested that the m^6^A deposition in HDAC6 mRNA may modulate the assembly and elongation of cilia.

To investigate whether the m^6^A of HDAC6 was associated with the acetylation of α‐tubulin as well as the cilia length, dm^6^ACRISPR was performed to achieve the targeted demethylation of HDAC6 (Figure S5B, Supporting Information).^[^ ^32^ ^]^ To achieve the specific demethylation of HDAC6, two guide RNAs (gRNAs) around the A3678 (m^6^A site) of HDAC6 mRNA were designed (Figure 5C). The specificity of gRNAs was confirmed by measuring HDAC6 mRNA level after co‐transfecting the active Cas13b and gRNAs (Figure S5C, Supporting Information). After dm^6^ACRISPR treatments, which co‐transfected dCas13b‐ALKBH5 and gRNAs, the results revealed that the mRNA level of HDAC6 was comparable to that of the control group (Figure S5D, Supporting Information). However, either the m^6^A level at A3678 of HDAC6 verified by SELECT (Figure 5D) or the total m^6^A level of HDAC6 verified by m^6^A‐RIP‐qPCR (Figure S5E, Supporting Information) suggested that both gRNAs could substantially reduce the m^6^A modification of HDAC6 mRNA. Accordingly, both protein levels (Figure 5E) and translation efficiency (Figure 5F) of endogenous HDAC6 were significantly decreased after dm^6^ACRISPR treatments. When applying dm^6^ACRISPR on pmirGLO‐HDAC6‐CDS‐WT, our results showed that targeted demethylation of reporter genes significantly suppressed its translation efficiency (Figure 5G; Figure S5F, Supporting Information). This suppression may be due to the targeted demethylation of HDAC6 mRNA, which could significantly decrease its binding with YTHDF3 (Figure 5H). Together, the targeted demethylation of HDAC6 can manipulate its expression artificially.

Next, we examined whether targeted demethylation of HDAC6 is involved in the acetylation of α‐tubulin and cilia elongation. Immunofluorescent assay presented that the gRNAs of HDAC6 combined with dCas13b‐ALKBH5 could significantly increase the cilia length of cancer cells (Figure 5I). Next, the western blot assay proved that the acetylation level of α‐tubulin significantly increased after demethylation of HDAC6 by using dm^6^ACRISPR (Figure 5J). In line with the acetylated tubulin levels, the length of cellular cilia was significantly increased after the demethylation of HDAC6 (Figure 5K). It suggests that manipulating m^6^A modification in HDAC6 mRNA can modulate cilia elongation.

### 2.6 HDAC6 Participates in METTL3‐Regulated Malignancy of Cancer Cells

**Figure 6:**
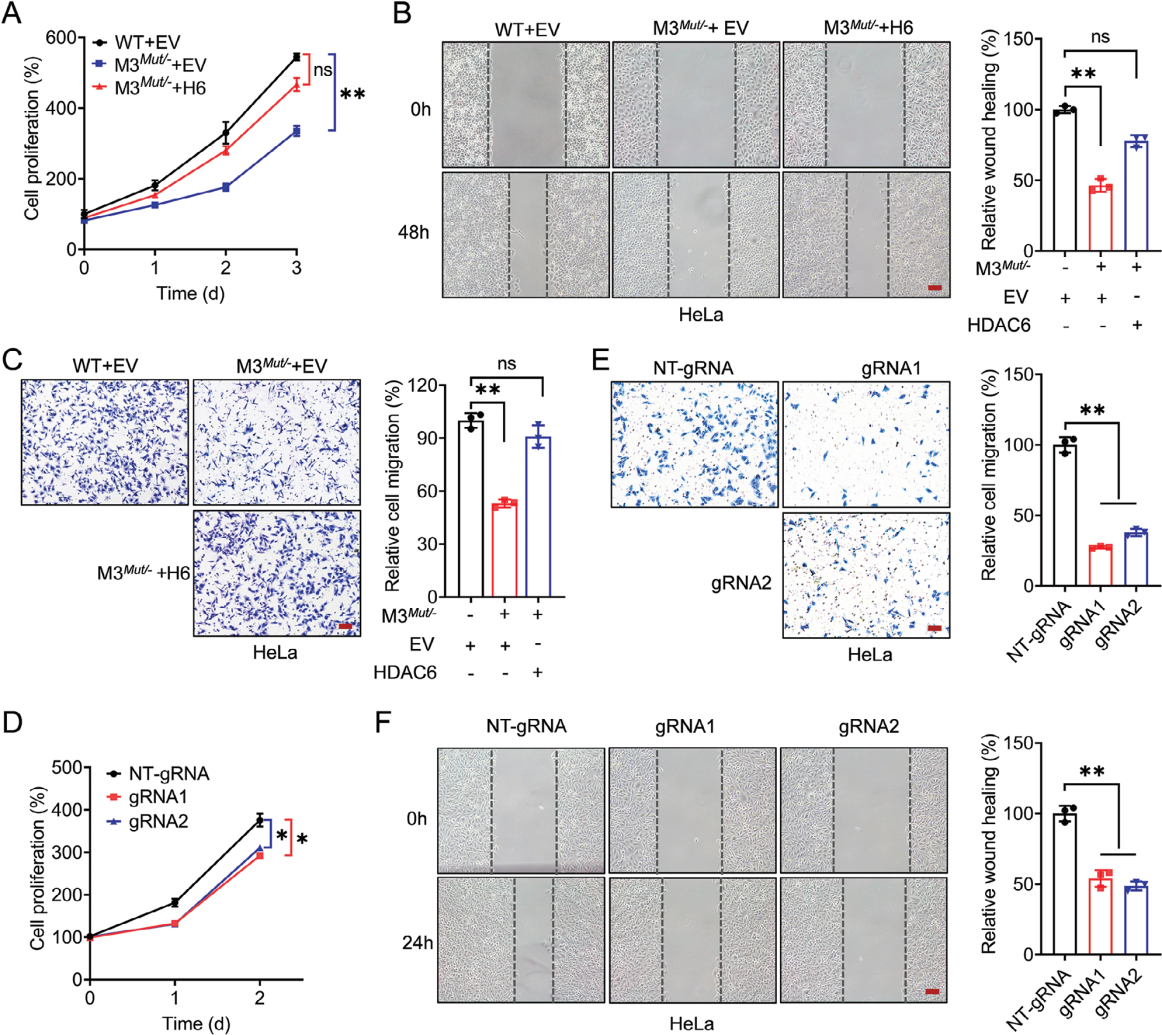
HDAC6 participates in METTL3‐regualted malignancy of cancer cells. A–C). The relative cell proliferation A), migration B), and invasion C) of WT or METTL3*^Mut/‐^* HeLa cells transfected with empty vector (EV, pcDNA3.1) or HDAC6 constructs (pcDNA3.1‐HDAC6‐3 × HA) (Scale bar,100 µm). D–F). The relative cell proliferation D), migration E), and invasion F) of HeLa cells transfected with dCas13b‐ALKBH5 combined with gRNA negative control or gRNA1/2, respectively, for 24 h (Scale bar,100 µm). Data are presented as mean ± SD from three independent experiments. *p < 0.05, **p < 0.01, ns, not significant, by Student's *t*‐test between two groups, and by one‐way ANOVA followed by Bonferroni's test for multiple comparisons.

Previous reports have suggested that METTL3 promotes the development of cervical cancer.^[^ ^33^ ^]^ We thus hypothesize that HDAC6 participates in the METTL3‐regulated malignancy of cancer cells. After overexpression of HDAC6 in METTL3*^Mut/−^* HeLa cells, the inhibitory effect of METTL3 knockdown on cell proliferation returned to levels similar to those of the control group (**Figure** **6**A). Similar observations were obtained in SiHa cells (Figure S6A, Supporting Information). In addition, wound healing assays and transwell assays revealed that overexpression of HDAC6 in METTL3*^Mut/^* ^−^ cells promoted the cell migration (Figure 6B; Figure S6B, Supporting Information) and invasion (Figure 6C; Figure S6C, Supporting Information) abilities of METTL3 knockdown cells. Together, these results indicated that HDAC6 plays a role in the METTL3‐regulated malignancy of cervical cancer in vitro.

Since m^6^A modification is crucial for regulating HDAC6 expression, we wonder whether targeted demethylation of HDAC6 mRNA by dm^6^ACRISPR can inhibit the tumorigenesis of cervical cancer cells. We then applied the dm^6^ACRISPR with gRNAs specifically targeting m^6^A modification of HDAC6 mRNA. Cell proliferation assay showed that the cell proliferation capacity was distinctly decelerated after dm^6^ACRISPR treatments (Figure 6D). In addition, both the invasive ability by transwell invasion assays (Figure 6E) and migratory capacity proved by wound healing assay (Figure 6F) showed obvious inhibition upon demethylation of HDAC6. To sum up, our results indicated that targeted m^6^A modification of HDAC6 by dm^6^ACRISPR was sufficient to suppress the proliferation, migration, and invasion of cervical cancer cells in vitro.

### 2.7 METTL3/HDAC6 Axis Promotes in Vivo Progression of Cervical Cancer

**Figure 7:**
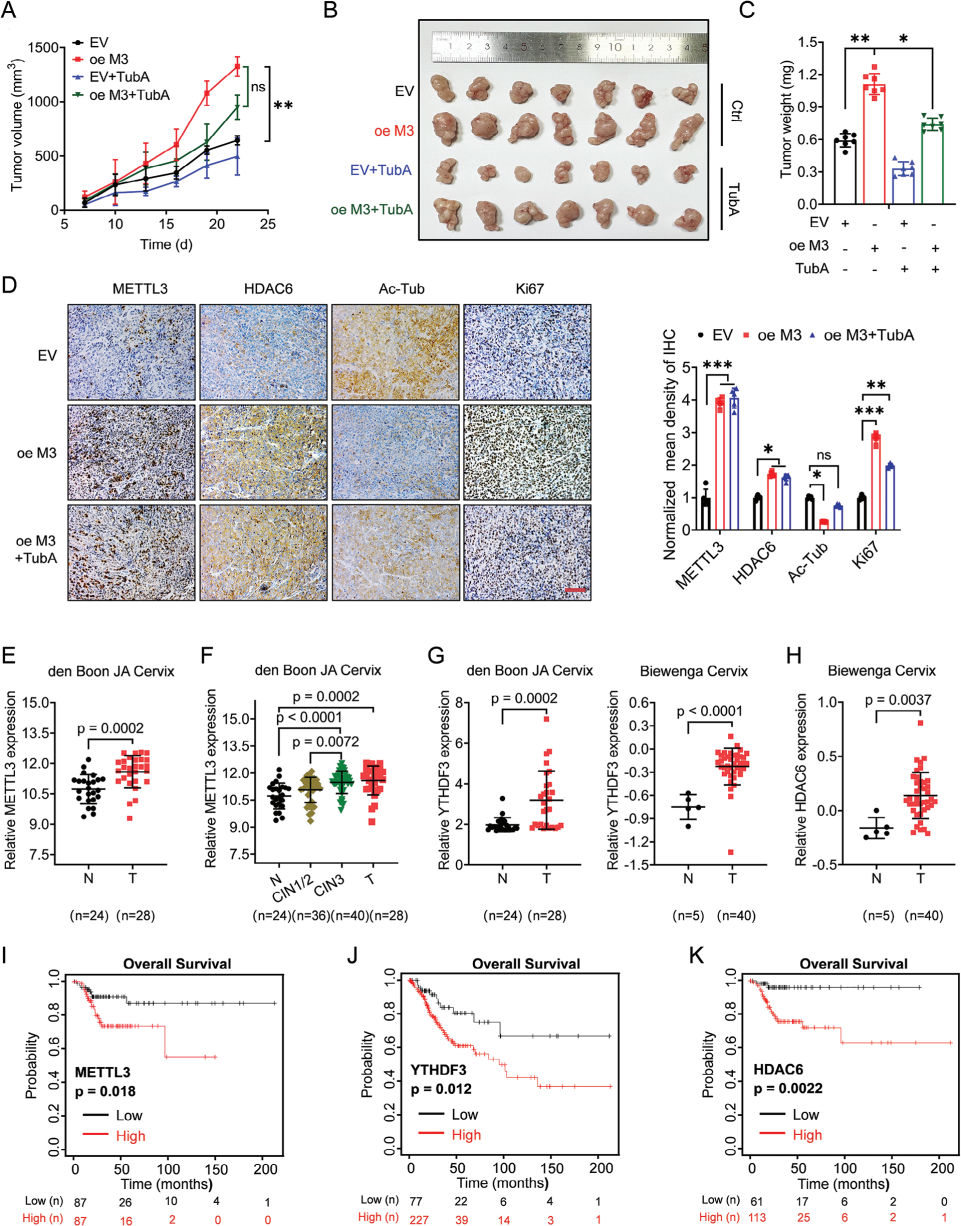
METTL3/HDAC6 axis promotes in vivo progression of cervical cancer. A) The tumor growth curves of xenografts established by stable overexpression of METTL3 in cervical cancer cells with the indicated treatments. B,C) The tumor volume B) and tumor weight C) of HeLa xenograft with the indicated treatments after exposure to Tubastatin A. Error bars are means ± SD, n = 7 independent repeats. P values were determined using two‐way ANOVA. D) IHC (METTL3, HDAC6, ac‐Tub, and ki67) ‐stained paraffin‐embedded sections obtained from control (EV) or METTL3 overexpression (oe M3) HeLa cells under described treatments. The scale bar is 100 µm. E–H) Relative mRNA expression of METTL3 E,F), YTHDF3 G) and HDAC6 H) in cervical tumor tissues and adjacent normal tissues in den Boon JA and Biewenga cervical cancers from GEO database. I–K) The survival curves of OS based on METTL3 I), YTHDF3 J), and HDAC6 K) expression in cervical cancer patients from the TCGA database. Data are presented as mean ± SD from three independent experiments. *p < 0.05, **p < 0.01, ns, not significant, by Student's *t*‐test between two groups, and by one‐way ANOVA followed by Bonferroni's test for multiple comparisons.

**Figure 8:**
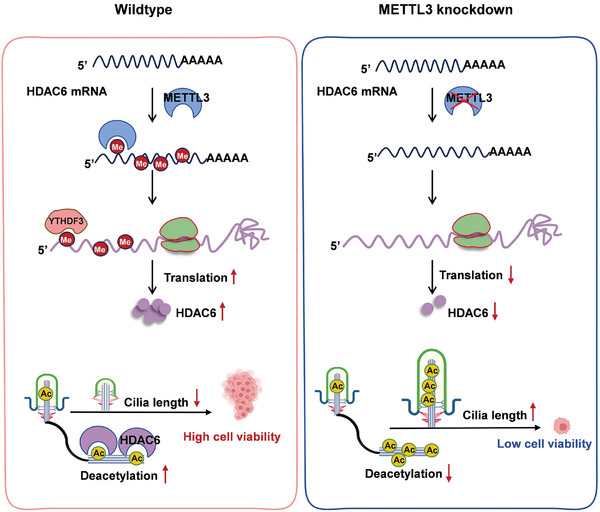
Working model of METTL3‐modulated cilia length of cervical cancer cells via HDAC6‐dependent deacetylation of axonemal α‐tubulin. As displayed in this model, METTL3 positively regulates the translation of HDAC6 in an m^6^A‐dependent manner by boosting the binding with YTHDF3, ultimately leading to enhanced protein expression. Additionally, the increased deacetylation of axonemal α‐tubulin induces the shortening of cilia length, thus accelerating the malignancy and proliferation of cancer cells. Correspondingly, visibly decreased m^6^A level of HDAC6 mRNA resulted from METTL3 knockdown attributed to significantly down‐regulated HDAC6 protein expression, which in return inhibits deacetylation of axonemal α‐tubulin, thus facilitating elongation of cilia length. And cells with increased cilia length exhibit lower cell viability and malignancy.

We further examined the cancer‐promoting effect of the METTL3/HDAC6 axis in cancer progression in vivo. HeLa cells with stable METTL3‐overexpression were established (Figure S7A, Supporting Information). The animal experimental procedure was presented in Figure S7B (Supporting Information), where HeLa cells were subcutaneously inoculated into the nude mice to establish tumor xenograft models. When tumor sizes reached ≈50 mm^3^, mice received intraperitoneal injections of HDAC6 inhibitor Tubastatin A (TubA) or vehicle every two days at a dose of 25 mg kg day^−1^ (Figure S7B, Supporting Information).^[^ ^34^ ^]^ After three weeks of treatment, mice tumors were collected and recorded. After analyzing the tumor sizes and weight, the results showed that overexpression of METTL3 significantly potentiated allograft growth of cervical cancer in vivo, which increased both tumor sizes and tumor weight (**Figure** **7**A–C), confirming the cancer‐promoting role of METTL3 in cancer progression. After inhibiting HDAC6 by TubA, it significantly suppressed the allograft growth of HeLa cells. Notably, TubA treatments distinctly abrogated METTL3‐induced cervical allograft growth in vivo. Consequently, immunohistochemistry (IHC) analysis showed HDAC6 and Ki67 levels were significantly elevated, while acetylated α‐tubulin levels were significantly reduced in xenograft tumor tissues overexpressing METTL3. Besides, pharmacological inhibition of HDAC6 sufficiently suppressed Ki67 levels and increased acetylated α‐tubulin levels (Figure 7D). Together, these results showed that both METTL3 and HDAC6 promote the progression of cervical cancer in vivo. Last, the tumor‐promoting effect of the METTL3/HDAC6 axis was verified with clinical data. The clinical online database of NCBI GEO (ncbi.nlm.nih.gov/geo) was used for analysis. As a result, in line with the oncogenic role of the METTL3/HDAC6 axis, higher expression of both METTL3 (Figure 7E,F), YTHDF3 (Figure 7G), and HDAC6 (Figure 7H) was observed in tumor tissues when compared to the matched adjacent normal tissues from cervical cancer patients. In addition, survival analysis showed that cervical cancer patients with increased expression of METTL3 (Figure 7I), YTHDF3 (Figure 7J), and HDAC6 (Figure 7K) represented an obviously unsatisfied overall survival (OS). Together, all these results implied the crucial function of the METTL3/HDAC6 axis in the pathogenesis of cervical cancer (**Figure** **8**).

## 3 Discussion

Increasing studies are rapidly advancing our understanding of the concrete mechanism of cilia in the mediation of cancer development.^[^ ^35^ ^]^ In terms of the antenna‐like and microtubule‐based structure of cilia, its capacity to integrate multiple environmental stimuli has been highly appreciated. Sometimes, effectors involved in ciliogenesis patterns have been elaborated to have dual and opposing roles in different tumor types and within tumor subtypes.^[^ ^2^ ^]^ In this study, we confirmed the presence of cilia in cervical cancer and the role of the METTL3/HDAC6 axis in modulating cilia length during cancer development. Until recently, the spotlight has been put on the role of METTL3 in a variety of diseases, especially in cancers.^[^ ^19^ ^]^ As displayed, the function of METTL3 has been extensively dissected. Nevertheless, the critical role of METTL3 in ciliogenesis and elongation, along with potential ciliation‐related targets, has not yet been highlighted. In our study, we found that inhibition of METTL3 could significantly restore ciliation in cervical cancer cells, which was characterized by increased elongation of cilia length via inhibiting HDAC6 expression. It has been confirmed by research that inhibiting HDAC6 can partially restore the cell phenotype with decreased cilia length.^[^ ^36^ ^]^ Moreover, HDAC6 has been extensively reported to induce ciliary resorption in cancer cells.^[^ ^35^, ^37^ ^]^ In our data, we found that overexpression of HDAC6 predominantly rescued stimulation implication of METTL3 depletion on the cilia assembly and elongation in HeLa and SiHa. Additionally, overexpression of HDAC6 also reversed the decreased cell proliferation and malignancy in METTL3 knockdown cells. Notably, this study introduces the pro‐tumorigenic role of m^6^A modification in cancer development from the perspective of cilia assembly and elongation for the first time, which is undoubtedly an interesting topic and provides a more theoretical basis for treatment strategies targeting cilia. Moreover, combining FDA‐approved HDAC6 inhibitors currently under clinical investigation with METTL3 inhibitors may offer a promising treatment strategy for cancers characterized by ciliary disorders, potentialy expanding therapeutic options.

The detailed mechanisms of RNA modification in fine‐tuning cilia structure and function were almost neglected until Narry Kim's group confirmed the integral role of m^6^A demethylase FTO (fat mass and obesity associated protein) in regulating motile ciliogenesis.^[^ ^38^ ^]^ As they elaborated, the repertoire of regulatory mechanisms of motile ciliogenesis was extensively broadened by unveiling its potential connection with dynamic m^6^A modification. In addition, Tianhua Zhou's group in 2022 further extended the repertoire of regulatory mechanisms of ciliogenesis by supporting the evidence of ALKBH3‐dependent m^1^A demethylation in ciliogenesis.^[^ ^39^ ^]^ ALKBH3 has been identified as a negative regulator of ciliogenesis in mammalian cells. Collectively, all these studies certify the obvious significance of RNA modification in ciliation, offering a novel highlight for further exploration.

The acetylation levels of proteins are dynamically determined by balancing transfer and removal of the acetyl group from the modified residues via enzymes with opposing activities: histone acetyltransferases (HATs) and HDACs. Importantly, a potential histone‐RNA crosstalk epigenetic mechanism in the oncogenesis has been elucidated.^[^ ^40^ ^]^ Previous studies have confirmed that Aurora A is phosphorylated and activated at the basal body during the initiation of ciliary disassembly, followed by phosphorylation and activation of HDAC6.^[^ ^41^ ^]^ Of note, ALKBH3 acts as a driver to manipulate ciliogenesis by targeting Aurora A in an m^1^A‐dependent manner.^[^ ^39^ ^]^ Emerging data has deciphered that HDAC6 deacetylates and destabilizes axonemal tubulin to facilitate ciliary disassembly.^[^ ^42^ ^]^ Importantly, the microtubule dynamics during cilia disassembly are predominately mediated by the deacetylation of tubulin installed by HDAC6. Currently, an increasing number of viewpoints have provided insights into the study of cilia length by focusing on the growth, stability, and post‐translational modifications of axonal microtubules.^[^ ^43^ ^]^ The post‐translational modifications of microtubules, including acetylation, detyrosination, glutamylation, and glycosylation, have emerged as critical drivers in regulating length, stability, and motility during ciliogenesis.^[^ ^43^, ^44^ ^]^ On the basis of our results, HDAC6 may participate in the METTL3‐regulated cilia length via deacetylating α‐tubulin. We observed that by upregulating the expression of HDAC6 in METTL3 knockdown cells, acetylated α‐tubulin levels were significantly inhibited, corresponding to the prolonged acetylated α‐tubulin signaling in cilia.

Collectively, our study sheds light on the previously neglected role and function of the m^6^A methyltransferase METTL3 in cilia elongation and cancer development. We identified HDAC6 as a critical regulator for METTL3‐meidated cilia elongation, which enhances the deacetylation of α‐tubulin to block cilia elongation. METTL3 deposits m^6^A in exon 25 of the HDAC6 mRNA to promote its translation via YTHDF3. METTL3‐mediated m^6^A modification weakens cilia elongation by enhancing HDAC6 expression, leading to a shortening of cilia length and ultimately accelerating cell proliferation and cancer progression. Additionally, while our study highlights the relationship between cilia length and the proliferation and migration capabilities of cervical cancer cells, it is essential to recognize that altered cilia length may also have implications for other cilia‐related functions, such as signal transduction and extracellular matrix sensing.^[^ ^2^, ^45^, ^46^ ^]^ These functions can influence cancer cell behavior, potentially contributing to tumor progression and metastasis. Future research should investigate these additional roles of cilia to gain a more comprehensive understanding of how cilia dynamics affect cancer biology.

## 4 Experimental Section

### Cell Line and Cell Culture

Human cancer HeLa, SiHa cells were purchased from the American Type Culture Collection (ATCC, Manassas, VA) and cultured in Dulbecco's modified Eagle medium (DMEM, GIBCO, Carlsbad, CA, USA) with 10% fetal bovine serum (FBS) and 1% penicillin/streptomycin (Invitrogen). Cell lines with stable knockdown or overexpression of targets were maintained in the medium containing the selected optimal puromycin.

### Plasmid, siRNA, shRNA, and Generation of Stable Cell Lines

The CDS of METTL3 and ALKBH5 were cloned into the PPB empty vector in our lab to generate plasmid over‐expression, while the PPB empty vector was used as the vector control for analysis. HDAC6 constructs (pcDNA3.1‐HDAC6‐3 × HA) and its vector control (pcDNA3.1) were both purchased from Miaolingbio (P9653, Wuhan, China). Meanwhile, METTL3 mutant DA (D395A) plasmids were constructed in the previous study.^[^ ^25^ ^]^ For YTHDF3 knockdown, two synthesized duplex RNAi oligos targeting human mRNA sequences were designed by Beijing Tsingke Biotech Co., Ltd. The most efficient one was selected for the following studies. A scrambled duplex RNA oligo (5′‐UUCUCCGAACGUGUCACGU) was used as RNA negative control (si‐NC). YTHDF3#1: 5′‐CAC CAA UGU CAG AUC CAU A‐3′; YTHDF3#2: 5′‐ CAG GCU UCA ACC AGA ACA A‐3′. Lipofectamine RNAiMAX (Invitrogen) was used for siRNA transfection. The working concentration for siRNA was 50 nM. To generate METTL3*^Mut/‐^* HeLa cells, the CRISPR‐Cas9 editing system was applied to achieve obvious METTL3 knockdown according to a previously published protocol.^[^ ^32^ ^]^ The stable cell lines sh‐NC and sh‐*METTL3* SiHa cells were produced through commercial virus packaging and screened with Dulbecco's modified Eagle medium (DMEM, GIBCO, Carlsbad, CA, USA) containing 10% FBS and 3 mg mL^−1^ puromycin for 3 weeks. Moreover, stable HeLa cells with overexpression of METTL3 were generated by virus packaging produced in the laboratory.

### Western Blot Analysis

Western blot analysis was performed as the protocol published previously.^[^ ^47^ ^]^ Briefly, total cell lysates were obtained with radio‐immunoprecipitation assay (RIPA) buffer containing 1 mM PMSF (Beyotime, China). Herein, protein concentration using the BCA protein assay kit (Thermo Fisher, USA) was quantified. About 20 µg total proteins were first separated by 8%/10% SDS‐PAGE gel, followed by electro‐transformation onto polyvinylidene fluoride (PVDF) membranes (Bio‐Rad, USA). Once the membranes were sealed with 5% nonfat milk in 1 x PBST at room temperature for 30 min, they were then incubated overnight with the corresponding primary antibody at 4 °C according to the dilution conditions indicated in the instruction manual. The primary antibodies contained in this study were as follows: anti‐METTL3 (15073‐1‐AP, proteintech, China); anti‐ALKBH5 (ab195377, Abcam, England); anti‐HDAC2 (12922‐3‐AP, proteintech); anti‐HDAC3 (10255‐1‐AP, proteintech); anti‐HDAC4 (17449‐1‐AP, proteintech); anti‐HDAC6 (7558S, CST, USA); anti‐HDAC8 (17548‐1‐AP, proteintech); anti‐HDAC9 (67364‐1‐Ig, proteintech); anti‐ARL13B (17711‐1‐AP, proteintech); anti‐IGF2BP1 (8482S, CST); anti‐IGF2BP2 (14672S, CST); anti‐IGF2BP3 (25864S, CST); anti‐YTHDF1 (17479‐1‐AP, proteintech); anti‐YTHDF2 (24744‐1‐AP, proteintech); anti‐YTHDF3 (25537‐1‐AP, proteintech); anti‐γ‐tubulin (ab179503, Abcam); anti‐α‐tubulin (A11126, Thermo Fisher Scientific, USA); anti‐acetyl‐α‐tubulin (32‐2700, Thermo Fisher Scientific); anti‐YTHDC2 (27779‐1‐AP, proteintech); HNRNPA2B1(14813‐1‐AP, proteintech); The bands results were analyzed by using ImageJ software (National Institutes of Health, Bethesda, MD, USA).

### RNA‐Extraction and Real‐Time PCR

Total RNA was isolated using TRIzol Reagent (Agbio, AG21102, China) and reversed by Evo M‐MLV (Agbio, AG11706, China). qRT‐PCR was determined with SYBR Green II (Agbio, AG11701, China) using CFX Manager 3.1 (Bio‐Rad, USA) as recommended by the manufacturer's protocol. The primers used are listed in Table S1 (Supporting Information). GAPDH was used as a control for normalization. The relative gene expression levels were calculated using the 2^−ΔΔCT^ method.

### Wound Healing Assay

Cells were seeded and cultured until a 90% confluent monolayer was formed. Cells were then scratched with a sterile pipette tip and subjected to treatments in the FBS‐free medium as indicated in the text. The migration distances of cells into the scratched area were measured in randomly chosen fields under a microscope.

### Immunofluorescence Analysis

For cilia staining, cells grown on coverslips were incubated in media without serum for 3 days to induce cilia assembly. After starvation for 3 days was performed, the cells were then put on ice for 30 min to label cilia, followed by the fixation with 100% prechilled methanol at −20 °C for 30 min. The following procedure was conducted according to standard protocol. Briefly, after washing with PBS 3 times, the cells were blocked with 5% BSA in 0.1% PBST (0.1% TritonX‐100 in phosphate‐buffered saline buffer) for 1 h at room temperature and stained with ciliary markers acetylated α‐tubulin (1:500, 32–2700, Thermo Fisher Scientific))/ARL13B (1:300, 17711‐1‐AP, proteintech) and/or γ‐tubulin (1:500, ab179503, Abcam) overnight at 4 °C. Fluorescence was monitored via inverted confocal laser microscopy (Olympus FV3000). Cilia length measurements were standardized to n = 30 for each group. The lengths of the cilia were calculated using ImageJ software. For cellular location analysis of HDAC6, cells were stained with primary antibodies of HDAC6 (1:200, 7558S, CST).

### SELECT qPCR

SELECT qPCR method was performed following Xiao's protocol with slight modifications.^[^ ^31^ ^]^ Total RNAs were first quantified by Qubit (Thermo Fisher Scientific) with Qubit RNA HS Assay Kit (Thermo Fisher Scientific). Next, 1500 ng of total RNA was mixed with 40 nM up and down primers and 5 µM dNTP in 17 µl 1 × CutSmart buffer (NEB). Then incubate the mixture under the conditions set in the following procedure: 90 °C for 1 min, 80 °C for 1 min, 70 °C for 1 min, 60 °C for 1 min, 50 °C for 1 min, and 40 °C for 6 min. The sample was further treated with 0.5 U SplintR ligase, 10 nM ATP, and 3 µl of 0.01 U Bst 2.0 DNA polymerase and incubated at 40 °C for 20 min, followed by denature at 80 °C for 20 min. And then, 20 µl qPCR reaction system containing 2 µl of the final reaction mixture, 2 × SYBR Green Master Mix (TaKaRa), and 200 nM SELECT primers (listed in Table S2, Supporting Information) was obtained. The qPCR procedure was set as follows: 95 °C, 5 min; (95 °C, 10 s; 60 °C, 35 s) × 40 cycles; 95 °C, 15 s; 60 °C, 1 min; 95 °C, 15 s; 4 °C, maintain. The results were calculated by normalizing the Ct values of samples to their corresponding control Ct values. All assays were performed with three independent experiments.

### Design of the Guide RNAs

mRNA sequences of all isoforms of target genes were subjected to alignment analysis to identify the common regions, which acted as targeting candidates for gRNA design. gRNAs targeting the CDS region of PDK4 were designed, and all designed gRNAs were subject to MEGABLAST (https://blast.ncbi.nlm.nih.gov/Blast.cgi) to avoid mismatching to unexpected mRNA in the human genome. The sequences of gRNAs were: gRNA1, 5′‐ GGTCCTTCTCTGTCTTCTACCTTCATGACCCGTAAGCTGC‐3′; gRNA2, 5′‐CTTCACATCTAGGAGAGCCTGGTGGTGGACATAG‐3′.

### m^6^A‐RNA Immunoprecipitation (MeRIP)‐qPCR

1 µg m^6^A or IgG antibody was incubated with Protein G Magnetic beads in 1x Reaction buffer (150 mM NaCl, 10 mM Tris‐HCl, pH 7.5, 0.1% NP‐40 in nuclease‐free H_2_O) at 4 °C for 3 h, followed by incubation with 200 µg extracted RNA at 4 °C for 3 h. Incubation of RNA‐antibody‐conjugated beads with 100 µl Elution Buffer (75 nM NaCl, 50 nM Tris‐HCl, pH 7.5, 6.25 nM EDTA, 1% (w/v) SDS, 20 mg ml^−1^ Proteinase K) for 30 min at room temperature was used to elute the bound RNAs. The eluted RNA was extracted by phenol: chloroform method followed by ethanol precipitation. Isolated m^6^A‐RIP RNA was reverse‐transcribed and quantification by qPCR. IP enrichment ratio of a transcript was calculated as the ratio of its amount in IP to that in the input yielded from the same amounts of cells.

### RNA Immunoprecipitation (RIP) RT‐PCR

The cell sample was firstly washed with prechilled PBS three times before collection, followed by irradiation with 400 mJ cm^2^ at 254 nm by Stratalinker on ice. 400 µl of high salt lysis buffer composed of 300 mM NaCl, 0.2% NP‐40, 20 mM Tris‐HCl PH 7.6, 0.5 mM DTT, protease inhibitor cocktail (1 tablet/50 ml), and RNase inhibitor (1:200) was used. Herein, lysate was centrifuged at 12 000 × g for 10 min to obtain cell supernatants. The above cell supernatants were then incubated with their corresponding Magnetic beads pre‐coated with 4 µl targeted antibodies or mouse IgG (NEB, USA) at 4 °C overnight. The beads containing immunoprecipitated RNA‐protein complex were incubated with proteinase K to exclude protein interference. Last, obtained RNAs were isolated by TRIzol methods and examined by RT‐qPCR with the normalization to input.

### Polysome Profiling

Polysome profiling was performed according to a procedure described previously.^[^ ^48^ ^]^ Briefly, METTL3 knockdown or control cells were first treated with 100 mg mL^−1^ CHX for 5 min at 37 °C before harvest to inhibit translation. The cell samples were obtained with lysis buffer (140 mM NaCl, 5 mM MgCl_2_, 10 mM Tris HCl (pH 8.0) containing 1% Triton X‐100, 0.5% sodium deoxycholate, 0.4 U mL^−1^ RNase inhibitor, 20 mM DTT, 0.1 mg mL^−1^ CHX, 10 mM RVC, and 0.1% cocktail. Next, the cell extracts were layered onto a sucrose gradient solution of 5% – 50% and centrifuged at 170 000 g in a Beckman SW‐41Ti rotor for 2 h at 4 °C to obtain different fractions in their corresponding sucrose gradient solution. Ribosomal fractions in each sucrose gradient were isolated by gradient profiling Triax (Biocomp Instruments), and then isolated d by Trizol to extract RNAs in each polysome fraction. The corresponding RNA concentrations were determined using the Qubit RNA HS Assay Kit (Thermo Fisher Scientific) for qRT‐PCR analysis.

### Cell Proliferation Assay

Cells were seeded in 96‐well plates at 1 × 10^4^ cells per well in a 10% FBS‐supplemented medium. After treatment as indicated conditions, cell proliferation was evaluated using the CCK‐8 cell viability assay system according to the manufacturer's protocol. A microplate reader was used to measure the absorbance at 450 nm.

### In Vitro Invasion Assay

The transwell assay was carried out using CytoSelect 24‐well cell invasion assay kits. Briefly, polycarbonate filters (8‐mm pore size, Corning) coated with 50% Matrigel (BD Biosciences, Bedford, MA, USA) were utilized to separate the upper and lower chambers. 200 µl of 5 × 10^4^ cell suspensions under the described treatments were added into the upper chamber, while 600 µl medium supplemented with 10% FBS was added to the lower chamber and acted as a chemotactic reagent. After 48h incubation, the cell samples in the lower chamber were then fixed, stained, and counted under an upright microscope (5 fields per chamber).

### Protein Stability Assay

WT or METTL3*^Mut/−^* HeLa cells were incubated with cycloheximide (CHX, final concentration of 50 µg ml^−1^) under described time periods. The protein stability was assessed by determining the expression of HDAC6 by western blot analysis.

### Subcellular Fractionation

Subcellular fractionation was carried out according to a previous study.^[^ ^49^ ^]^ Briefly, the cytoplasmic and nuclear fractions were obtained by NE‐PER nuclear and cytoplasmic extraction reagents (Pierce, Rockford, IL, USA). Subsequently, the cytoplasmic and nuclear RNA were extracted by adding TRIzol reagent for qRT‐PCR according to the published procedure.^[^ ^28^ ^]^ HDAC6 mRNA in cytoplasmic and nuclear RNA was examined by real‐time PCR, where GAPDH and HPRT served as controls for normalization in the cytoplasmic and nuclear fractions, respectively.

### Luciferase Reporter Assay

The CDS region of HDAC6 was subcloned into the dual‐luciferase vector pmirGLO (Promega, USA) to construct the Dual‐Luciferase Reporter Assay System. Briefly, luciferase activity was measured by Dual‐Luciferase Reporter Gene Assay Kit (Beyotime, China) according to the manufacturer's instructions. The firefly luciferase activity values were normalized to the Renilla luciferase activity values that evaluate expression level. Herein, the translation efficiency was examined by a relative value of F‐luc divided by R‐luc. Experiments were performed three times with similar results.

### In Vivo Experiments

All animal experimentation was carried out in accordance with and approved by Zhongshan School of Medicine Policy on Care and Use of Laboratory Animals. Female BALB/c nude mice (four weeks old) with initial mean body weights ranging between 18 and 20 g were obtained from Sun Yat‐sen University (Guangzhou, China) Animal Center and housed in sterile filter‐capped microisolator cages and provided with sterilized food and water. To construct the subcutaneous xenograft model, ≈1 × 10^6^ HeLa cells suspended in 50% Matrigel in DMEM were subcutaneously injected into the right flanks of the mice. All these mice were randomly divided into four groups (each containing 7 mice). When the tumor size reached ≈50 mm^3^, mice received intraperitoneal injections of TubA or vehicle every two days at a dose of 25 mg kg day^−1^ for three consecutive weeks, as previously described.^[^ ^34^, ^50^ ^]^ Once administration finished, mice were euthanized and the tumors under different treatments were harvested. Moreover, tumor size and weights were monitored and calculated for further analysis. Herein, tumor growth was monitored every three days. The tumor volume was calculated using the following formula: 1/2 × larger diameter × (smaller diameter)^2^.

### Immunohistochemistry (IHC) Analysis

The xenograft slides were deparaffinized and rehydrated through an alcohol gradient, followed by antigen retrieval with sodium citrate buffer. Tumor sections were first blocked with 5% normal goat serum (vector) with 0.1% Triton X‐100 and 3% H_2_O_2_ in PBS for 60 min at room temperature, followed by incubation with corresponding primary antibodies at 4 °C overnight. IHC staining was conducted with horseradish peroxidase (HRP) conjugates using DAB detection. Images were taken with a Nikon microscope.

### Database (DB) Analysis

The Kaplan Meier database has been applied to evaluate the prognostic value of targets (METTL3, YTHDF3, and HDAC6) in clinics based on their OS in cancer individuals. The difference between survival curves was determined by the log rank test; *p* <  0.05 was considered statistically significant. The data were analyzed with a Pearson chi‐square test. Analyze the differences in mRNA levels of METTL3, YTHDF3, and HDAC6 between cancer and normal cervical cancer tissues obtained from the GEO database (GSE7410 and GSE63514).

### Statistical Analysis

Data were reported as mean ± SD from at least three independent experiments. For statistical analysis, two‐tailed unpaired Student's *t*‐test between two groups and by one‐way or two‐way ANOVA followed by Bonferroni's test for multiple comparisons was performed. All statistical tests were two‐sided. Data analysis was carried out using SPSS 16.0 for Windows. A *p*‐value of <  0.05 was considered to be statistically significant. *p <0.05, **p < 0.01, ***p < 0.001; ns, not significant.

## Conflict of Interest

The authors declare no conflict of interest.

## Author Contributions

Y.R. and H.Z. contributed equally to this work. H.S.W., Z.G.L., K.Z., and J.X.L. designed and initiated the study. Y.L.R., H.S.Z., K.N.Y., C.L.G., and S.Y.Q. performed experiments. X.S.W., W.F.Y., G.A., C.L.G., and J.X.P. helped design the study and interpret the data. Y.L.R., J.X.L., J.M.H., and H.S.W. wrote and revised the manuscript.

## Supporting information



Supporting Information

## Data Availability

The data that support the findings of this study are available on request from the corresponding author. The data are not publicly available due to privacy or ethical restrictions.
