# Supplementary material for: N6‐Methyladenosine Regulates Cilia Elongation in Cancer Cells by Modulating HDAC6 Expression
Source: Adv Sci (Weinh). 2024 Nov 13;12(2):2408488. doi: 10.1002/advs.202408488 (PMC11727115; doi:10.1002/advs.202408488)
Supplement: Supplementary file 1 — Supporting Information [file ADVS-12-2408488-s001.docx]

**Supporting Information for**

**N^6^-methyladenosine Regulates Cilia Elongation in Cancer Cells by Modulating HDAC6 Expression**

*Yalan Rui ^1#^, Haisheng Zhang ^1#^, Kangning Yu ^1^, Shiyao Qiao ^1^, Chenglin Gao ^1^, Xiansong Wang ^1^, Weifeng Yang ^1^, Gholamreza Asadikaram ^2^, Zigang Li ^3^, Kun Zhang ^4^, Jianxin Peng ^5^, Jiexin Li ^1^*, Junming He ^5^*, Hongsheng Wang ^1^**

**Supplementary Figures and Figure legends**

**Figure S1.**

**
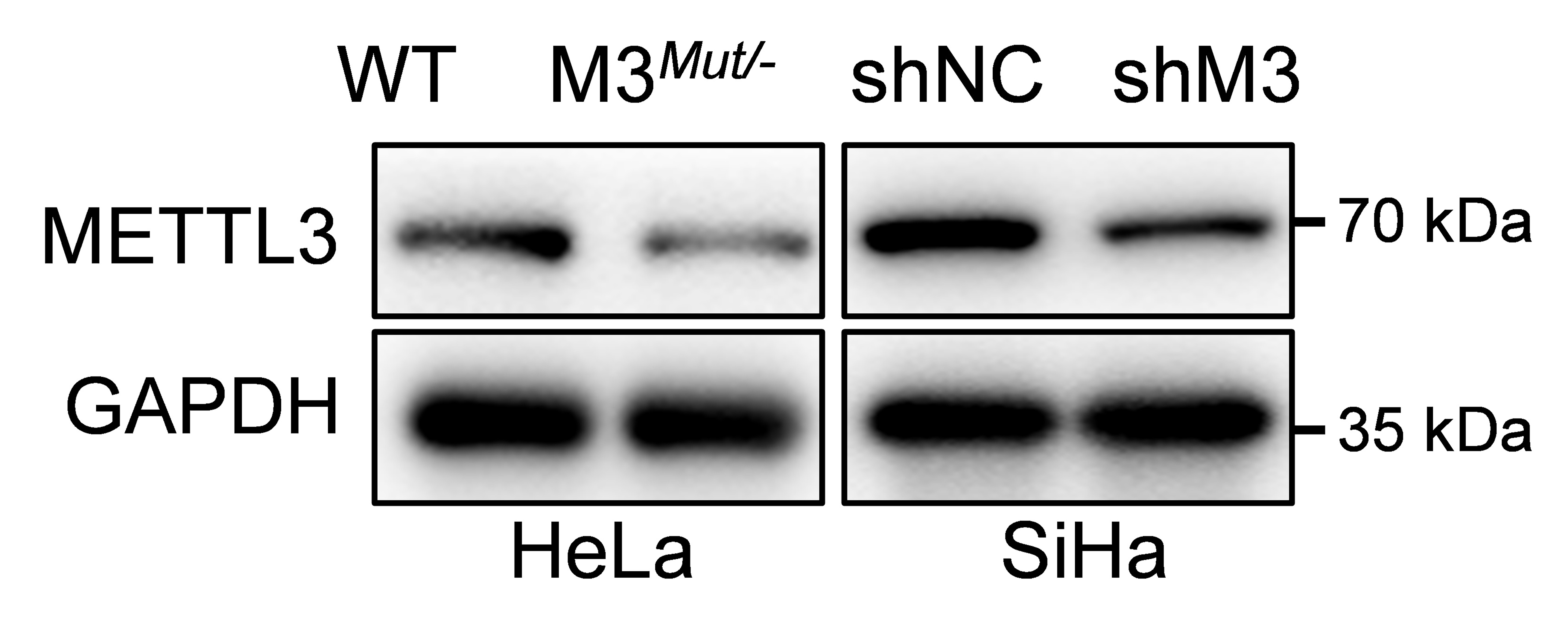
**

**Figure S1.** METTL3 suppresses the ciliary elongation in cervical cancer cells.

Expression levels of METTL3 in WT or METTL3*^Mut/-^* HeLa cells or shNC/shMETTL3 SiHa were examined by western blot analysis.

Data are presented as mean ± SD from three independent experiments. **p*<0.05, ***p*<0.01, ****p*<0.001, ns, not significant, by Student’s *t*-test between two groups and by one-way ANOVA followed by Bonferroni’s test for multiple comparisons.

**Related to Figure 1.**

**Figure S2.**





**Figure S2.** HDAC6 is participated in the METTL3-regulated cilia elongation.

1. The protein levels of class I (HDAC 2, 3 and 8), class IIa (HDAC4) and class IIb (HDAC6) were systematically examined in shNC/shMETTL3 SiHa cells by western blot analysis.
2. The protein expression of HDAC6 in SiHa cells transfected with vector control or ALKBH5 plasmid was measured by western blot analysis.
3. The protein levels of HDAC6 in SiHa WT cells treated with different concentrations of STM2457 (1.5, 2μg/ml) were measured by western blot analysis.
4. The relative expression of HDAC6 in WT or METTL3*^Mut/-^* HeLa cells transfected with vector control (pcDNA3.1) or its HDAC6 constructs (pcDNA3.1-HDAC6-3×HA) for 48 h was measured by western blot analysis.
5. Representative confocal images of WT or METTL3*^Mut/-^* HeLa cells were visualized by using anti-HDAC6 antibody (green) (Scale bar, 5 μm).
6. The relative ratio of Ac-Tub/α-Tub in shNC/shMETTL3 SiHa cells transfected with vector control (pcDNA3.1) or HDAC6 constructs (pcDNA3.1-HDAC6-3×HA) for 48 h was measured by western blot analysis.
7. The acetylated α-tubulin length of cilia in in SiHa shNC/shMETTL3 cells was examined. Primary cilia were visualized by immunofluorescences using anti-ac-tubulin antibody (green) and the basal body using γ-tubulin antibody (red), and nuclei were stained with DAPI (blue). The acetylated α-tubulin length was measured and presented as bar graphs (*right*) (Scale bar, 5 μm).
8. The length of cilia in SiHa cells treated with DMSO or STM2457 (2μg/ml) for 48 h was examined. Primary cilia were visualized by immunofluorescences using anti-ac-α-tubulin antibody (green) and the basal body using γ-tubulin antibody (red), and nuclei were stained with DAPI (blue). The acetylated α-tubulin length was measured and presented as bar graphs (*right*) (Scale bar, 5 μm).

Data are presented as mean ± SD from three independent experiments. **p*<0.05, ***p*<0.01, ****p*<0.001, by Student’s *t*-test between two groups, and by one-way ANOVA followed by Bonferroni’s test for multiple comparisons.

**Related to Figure 2.**

**Figure S3.**

**
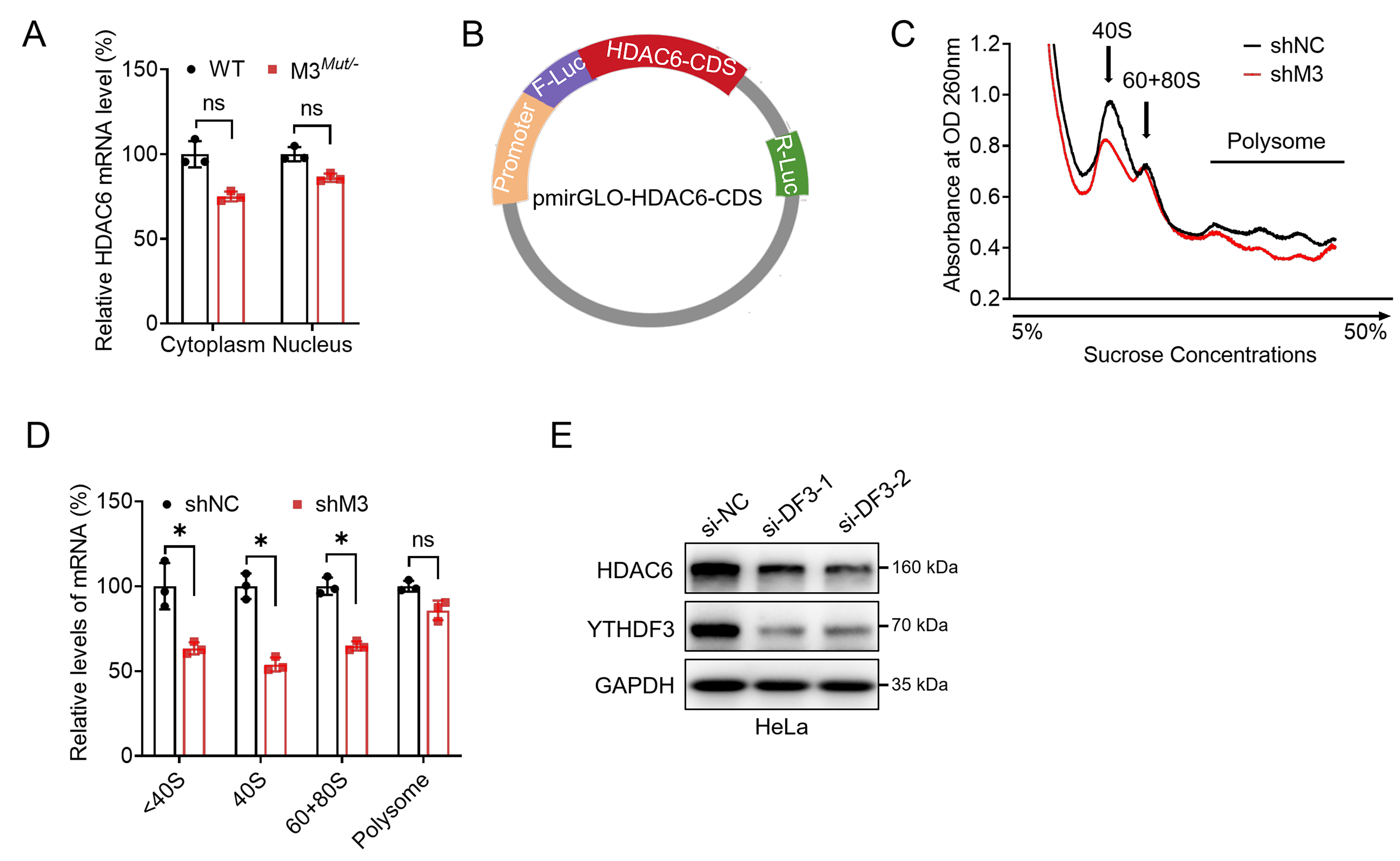
**

**Figure S3.** METTL3 regulates the translation of HDAC6 via YTHDF3.

1. The relative levels of nuclear versus cytoplasmic HDAC6 mRNA in WT or METTL3*^Mut/-^* HeLa cells were measured.
2. Schematic of pmirGLO-HDAC6-CDS-WT reporter.
3. The polysome profiling of shNC/shMETTL3 SiHa cells was analyzed.
4. The mRNA levels of HDAC6 in non-ribosome portion (<40S), 40S, 60S, 80S, and polysome fractions in shNC/shMETTL3 SiHa cells were checked by qRT-PCR.
5. Expression levels of YTHDF3 in HeLa WT cells transfected with si-YTHDF3-1/-2 were detected by western blot assay.

Data are presented as mean ± SD from three independent experiments. **p*<0.05, ns, not significant, by Student’s *t*-test.

**Related to Figure 3.**

**Figure S4.**

**
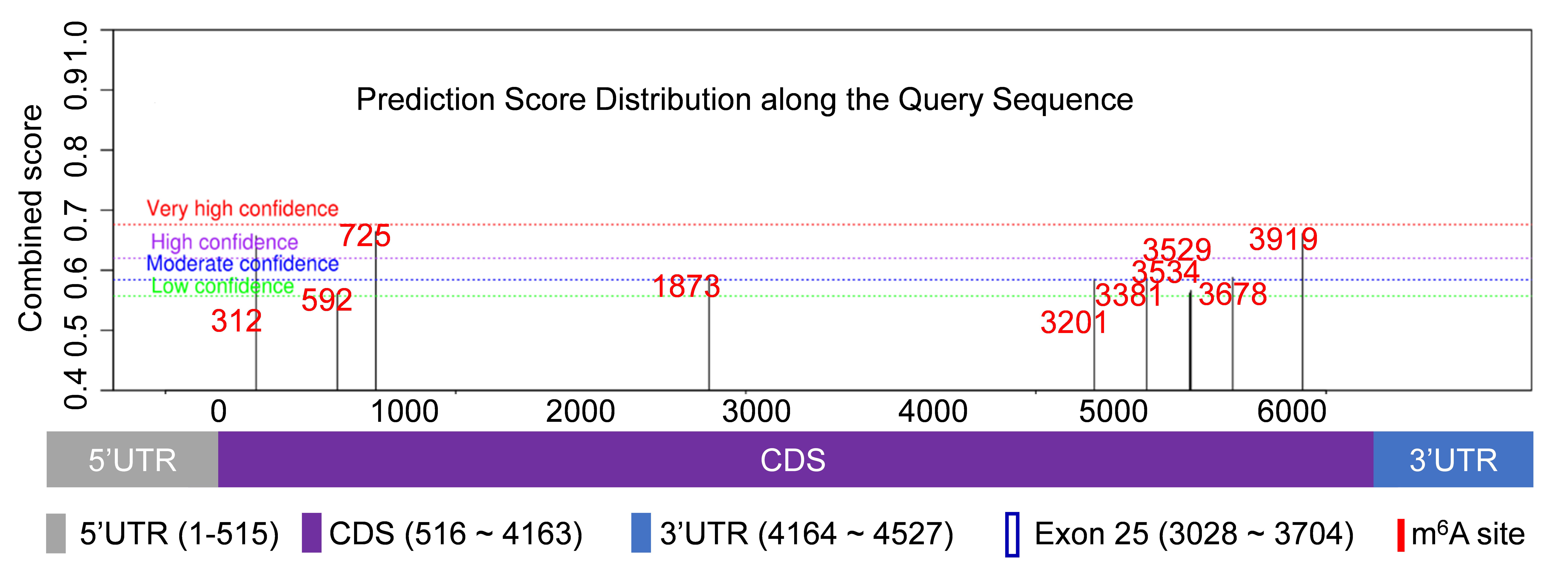
**

**Figure S4.** Methylation sites of HDAC6 involved in m^6^A regulated expression of HDAC6.

The predicted m^6^A sites in HDAC6 mRNA from the m^6^A sites predictor SRAMP.

**Related to Figure 4.**

**Figure S5.**


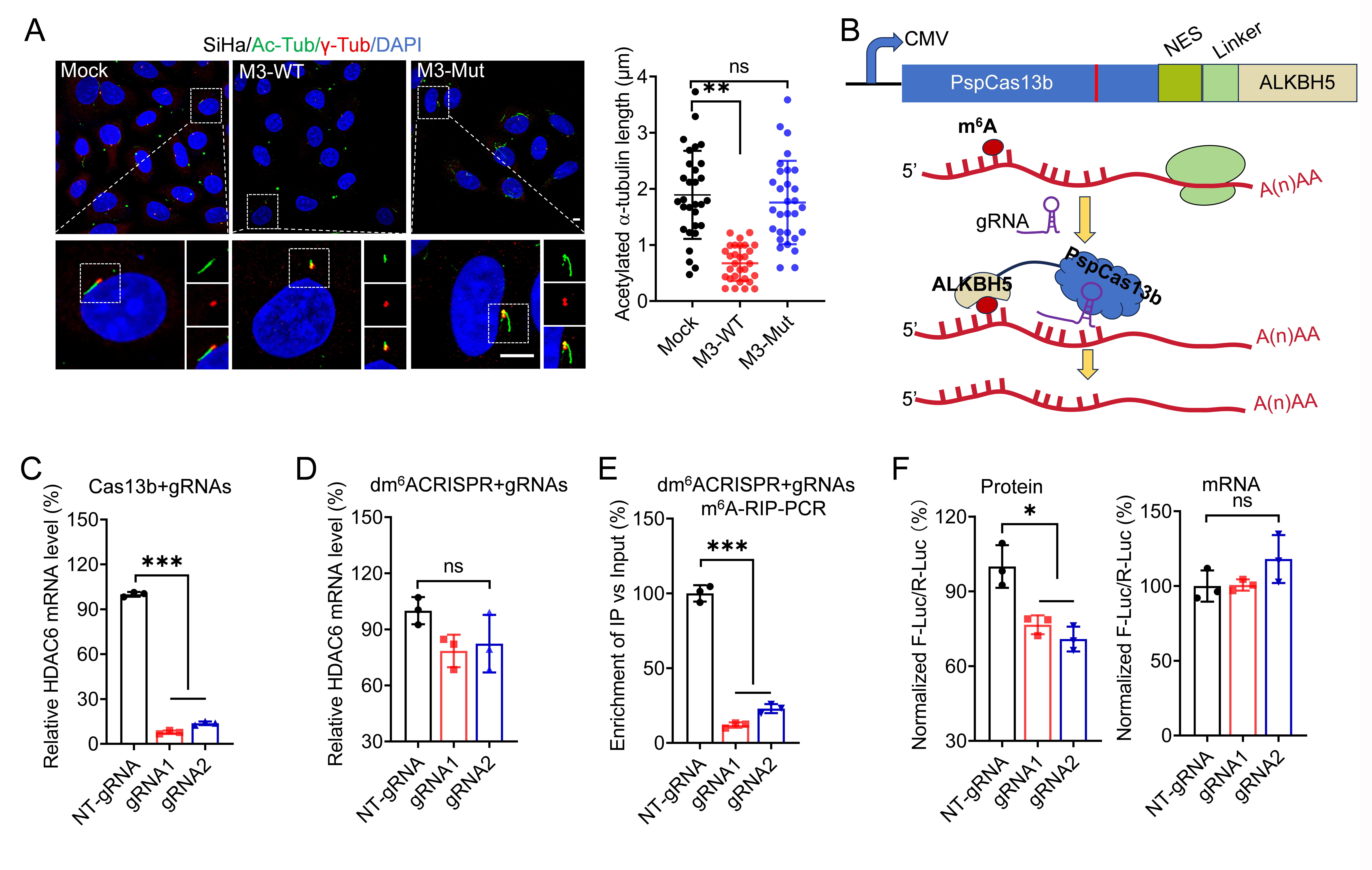


**Figure S5.** Targeted demethylation of HDAC6 by dm^6^ACRISPR disturbs cilia elongation.

1. SiHa wildtype cells were transfected with PPB-METTL3 (M3-WT), PPB-METTL3 mutant (M3-mut) or the empty vector PPB (Mock), respectively. Primary cilia were visualized by immunofluorescences using anti-ac-tubulin antibody (green) and the basal body using γ-tubulin antibody (red), and nuclei were stained with DAPI (blue). The acetylated α-tubulin length was measured and presented as bar graphs (*right*) (Scale bar, 5μm).
2. Schematic illustration of the domain organization of the dCas13bALKBH5 expression cassette and site-specific RNA targeting using dCas13b-guided fusion proteins.
3. The mRNA expression of HDAC6 in HeLa cells transfected with Cas13b combined with gRNA negative control or gRNA1/2, respectively, for 24 h.
4. The mRNA expression of HDAC6 in HeLa cells transfected with dCas13b-ALKBH5 combined with gRNA negative control or gRNA1/2, respectively, for 24 h.
5. m^6^A-RIP–PCR analysis of HDAC6 mRNA in HeLa cells transfected with dCas13b-ALKBH5 combined with gRNA negative control or gRNA1/2, respectively, for 24 h.
6. WT or METTL3*^Mut/-^* HeLa cells were co-transfected with pmirGLO-HDAC6-CDS-WT reporter and dCas13b-ALKBH5 combined with gRNA negative control or gRNA1/2, for 24 h. The protein and mRNA level of HDAC6 were determined.

Data are presented as mean ± SD from three independent experiments. **p*<0.05, ***p*<0.01, ****p*<0.001, by Student’s *t*-test between two groups, and by one-way ANOVA followed by Bonferroni’s test for multiple comparisons.

**Related to Figure 5.**

**Figure S6.**


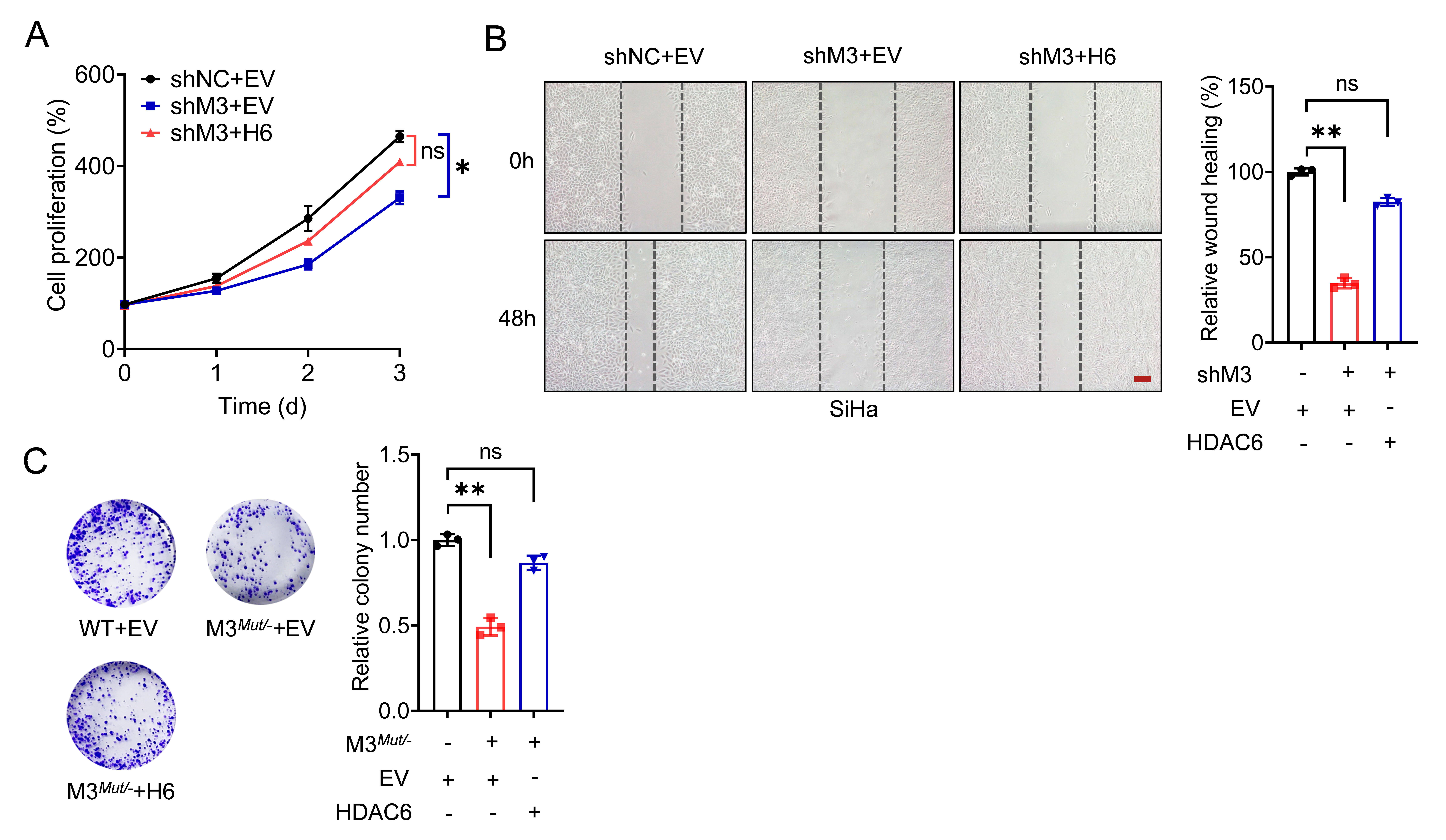


**Figure S6.** HDAC6 participates in METTL3-regualted malignancy of cervical cancer in vitro*.*

(A-B) The relative cell proliferation (A) and migration (B) of shNC/shMETTL3 SiHa cells transfected with vector control (pcDNA3.1) or its HDAC6 constructs (pcDNA3.1-HDAC6-3×HA) (Scale bar, 100 μm).

(C) The colony‑formation assays were performed to evaluate the proliferation of WT or METTL3*^Mut/-^* HeLa cells transfected with vector control (pcDNA3.1) or its HDAC6 constructs (pcDNA3.1-HDAC6-3×HA). Relative colony number was recorded (*left*) and analyzed quantitatively (*right*).

Data are presented as mean ± SD from three independent experiments. **p*<0.05, ***p*<0.01, by Student’s *t*-test between two groups, and by one-way ANOVA followed by Bonferroni’s test for multiple comparisons.

**Related to Figure 6.**

**Figure S7.**


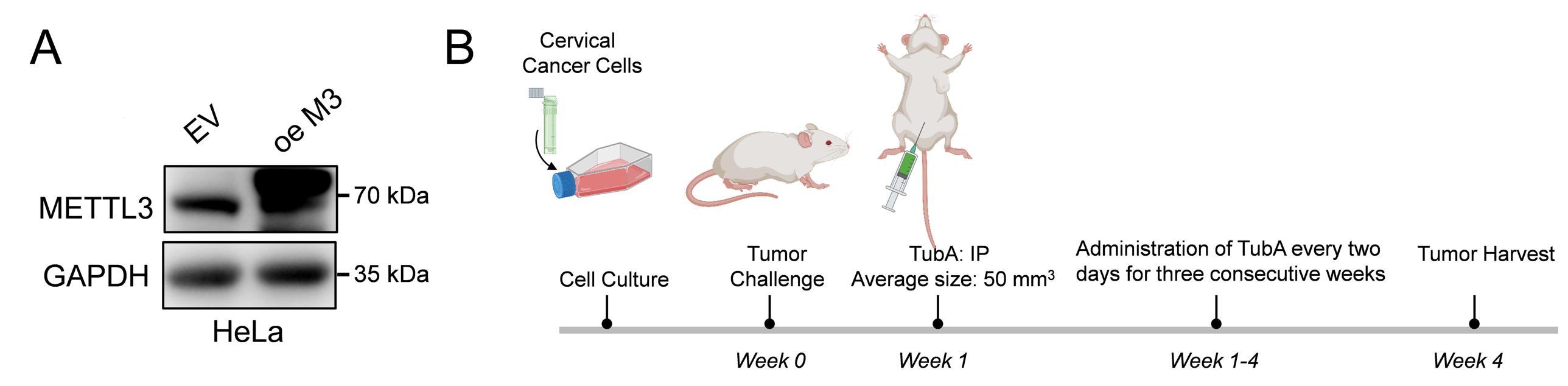


**Figure S7.** METTL3/HDAC6 axis promotes in vivo progression of cervical cancer.

(A) The overexpression of METTL3 was confirmed by western blot analysis.

(B) Schematic of drug treatments in mouse model. Daily dosing began when the tumor sizes reached about 50 mm^3^. Female BALB/c nude mice received intraperitoneal injections of HDAC6 inhibitor, Tubastatin A (TubA), or vehicle every two days at a dose of 25 mg/kg/day. After three weeks of treatment, mice tumors were collected and recorded.

**Related to Figure 7.**

**Supplementary Tables**

**Table S1. Primers used in the present study**

| Gene | | Primer sequence (qRT-PCR) |
| --- | --- | --- |
| HDAC6 | | Forward: 5’- AAGAAGACCTAATCGTGGGACT -3’  Reverse: 5’- GCTGTGAACCAACATCAGCTC -3’ |
| GAPDH | | Forward: 5’- GCACCGTCAAGGCTGAGAAC -3’  Reverse: 5’- TGGTGAAGACGCCAGTGGA -3’ |
| METTL3 | | Forward: 5’- CTATCTCCTGGCACTCGCAAGA -3’  Reverse: 5’- GCTTGAACCGTGCAACCACATC -3’ |
| ALKBH5 | | Forward: 5’- CCAGCTATGCTTCAGATCGCCT -3’  Reverse: 5’- GGTTCTCTTCCTTGTCCATCTCC-3’ |
| 5’UTR-HDAC6 | | Forward: 5’- AGAGTAGAAGGGGCGGTGATTG -3’  Reverse: 5’- TTCCCTGGTCTTGCCCTTTC -3’ |
| 3’UTR-HDAC6 | | Forward: 5’- CCACTGCATCCCATCCTGAATATC -3’  Reverse: 5’- GAGTAGTGGTGCCCCTCAAGC -3’ |
| CDS-HDAC6 | | Forward: 5’- GTAGGAGGAGCTACACTGGC -3’  Reverse: 5’- GGAGTTTGGATCAGCTCGGTG -3’ |
| Exon 2 | | Forward: 5’-CCAGGATTCCACCACAACCA -3’  Reverse: 5’- ACCTCCGCTAGATTGGGGATAG -3’ |
| Exon 3 | | Forward: 5’- CGGCCAAGCAATGGAAGAAG -3’  Reverse: 5’- GCTGTCATCCCAGAGGCAATG -3’ |
| Exon 25 | | Forward: 5’- GGAAAGTCACCTCGGCATC -3’  Reverse: 5’- GGCTGCCTCAGAAATGGTCTG -3’ |
| Exon 27 | | Forward: 5’- CAGGCCATATTTTATGCTGTGAC -3’  Reverse: 5’- CTGATAGCAAGAGAGACACAC -3’ |
| HPRT | | Forward: 5’- TGACACTGGCAAAACAATGCA -3’  Reverse: 5’- GGTCCTTTTCACCAGCAAGCT -3’ |
| 18S rRNA | | Forward: 5’- CGGACAGGATTGACAGATTGATAGC -3’  Reverse: 5’- TGCCAGAGTCTCGTTCGTTATCG -3’ |
| Firefy-Luc | | Forward: 5’- GGTACTGTTGGTAAAGCCAC -3’  Reverse: 5’- CTCTTCATAGCCTTATGCAG -3’ |
| Renilla-Luc | | Forward: 5’- CAATGGGCAGGTGTCCACTC -3’  Reverse: 5’- GTTCTGGATCATAAACTTTC -3’ |
| Mutation plasmid | Primer sequence | |
| Exon25-Mut1 | Forward: AGTCCACTCCAGGCCAGCCTAACTCAGAGACAGCT  Reverse: AGCTGTCTCTGAGTTAGGCTGGCCTGGAGTGGACT | |
| Exon25-Mut2 | Forward: CCACCTCAGAGGAGCCTGTGGGAGGAGCC  Reverse: GGCTCCTCCCACAGGCTCCTCTGAGGTGG | |
| Exon25-Mut3 | Forward: GCCACACTGGCCCAGACTACGTCAGAGGAGGCTCCAG  Reverse: GACGTAGTCTGGGCCAGTGTGGCTCCCTCCATGGCTG | |
| Exon25-Mut4 | Forward: GGAGGGAGCCACACTGGACCAGCCTACGTCAG  Reverse: GGAGCCTCCTCTGACGTAGGCTGGTCCAGTG | |
| Exon25-Mut5 | Forward: ATTGGGAGTCTCAGGCCCTTGGAGCTAGGCA  Reverse: TGCCTAGCTCCAAGGGCCTGAGACTCCCAAT | |

**Table S2. Primers used for SELECT PCR**

| SELECT | Primer for SELECT PCR |
| --- | --- |
| HDAC6-X | Forward: 5’- TAGCCAGTACCGTAGTGCGTGCTGAGATTCGCTGCCTAGCTCCAAGG -3’ |
|  | Reverse: 5’phos- CCTGAGACTCCCAATCAGTGTACTGGGAGCAGAGGCTGAGTCGCTGCAT -3’ |
| HDAC6-N | Forward: 5’- TAGCCAGTACCGTAGTGCGTGGCTGCCTAGCTCCAAGGTCCTGAGAC -3’ |
|  | Reverse: 5’phos- CCCAATCAGTGTACTGGGAGATATCTGCAGAGGCTGAGTCGCTGCAT -3’ |
| q-PCR SELECT | Forward: 5’- ATGCAGCGACTCAGCCTCTG -3’  Reverse: 5’- TAGCCAGTACCGTAGTGCGTG -3’ |
